# From cold to hot: the immunomodulatory framework of Saikosaponin D in hepatocellular carcinoma therapy

**DOI:** 10.3389/fimmu.2026.1964907

**Published:** 2026-10-01

**Authors:** Xiaoyue Huang, Lin Liu, Meihui Qin, Runyue Liu

**Affiliations:** 1 College of Traditional Chinese Medicine, Hubei University of Chinese Medicine, Wuhan, China; 2 College of Acupuncture-Moxibustion and Orthopedics, Hubei University of Chinese Medicine, Wuhan, China

**Keywords:** cold tumor microenvironment, hepatocellular carcinoma, immune conversion, immunogenic cell death, Saikosaponin d, tumor-associated macrophages

## Abstract

**Background:**

Hepatocellular carcinoma (HCC) is the sixth most common cancer and the third leading cause of cancer-related mortality worldwide. Despite the clinical success of immune checkpoint inhibitors, a substantial proportion of patients exhibit primary resistance, largely attributable to the immunosuppressive “cold” tumor microenvironment (TME). Saikosaponin D (SSd), a triterpenoid saponin derived from Bupleurum falcatum L., has emerged as a multifaceted immunomodulator with potential to remodel the TME, yet its role in converting “cold” HCC to an immunologically responsive “hot” phenotype remains systematically unexplored.

**Objective:**

This review proposes a hypothesis-generating framework for saikosaponin D mediated cold-to-hot immune conversion in hepatocellular carcinoma. We synthesize preclinical evidence and integrate it into a dynamic temporal model to guide future research.

**Materials and methods:**

We systematically searched PubMed, EMBASE, Web of Science, and Cochrane Library from January 2020 to December 2025. Search terms included saikosaponin D, hepatocellular carcinoma, and immunomodulation. The search was broadened to solid tumor immunology due to limited hepatocellular carcinoma specific studies. Two investigators screened and extracted data. From 306 initial records, 139 publications were included.

**Results:**

Saikosaponin D modulates multiple signaling pathways including PI3K/AKT/mTOR, STAT3, HIF-1α, and TGF-β. It may promote immunogenic cell death, dendritic cell maturation, T-cell recruitment, macrophage repolarization, and metabolic remodeling. We propose a four-stage temporal model of immune conversion. Low oral bioavailability and missing orthotopic validation are key challenges that nanoformulation and organoid models may overcome.

**Conclusions:**

Saikosaponin D shows multi-target immunomodulatory potential as a microenvironment-priming agent. The current evidence is largely indirect and derived from non-hepatocellular carcinoma models. Future validation requires orthotopic studies, advanced nanoformulations, and formal synergy testing. This framework offers testable hypotheses rather than therapeutic claims.

## Graphical Abstract

**Figure:**
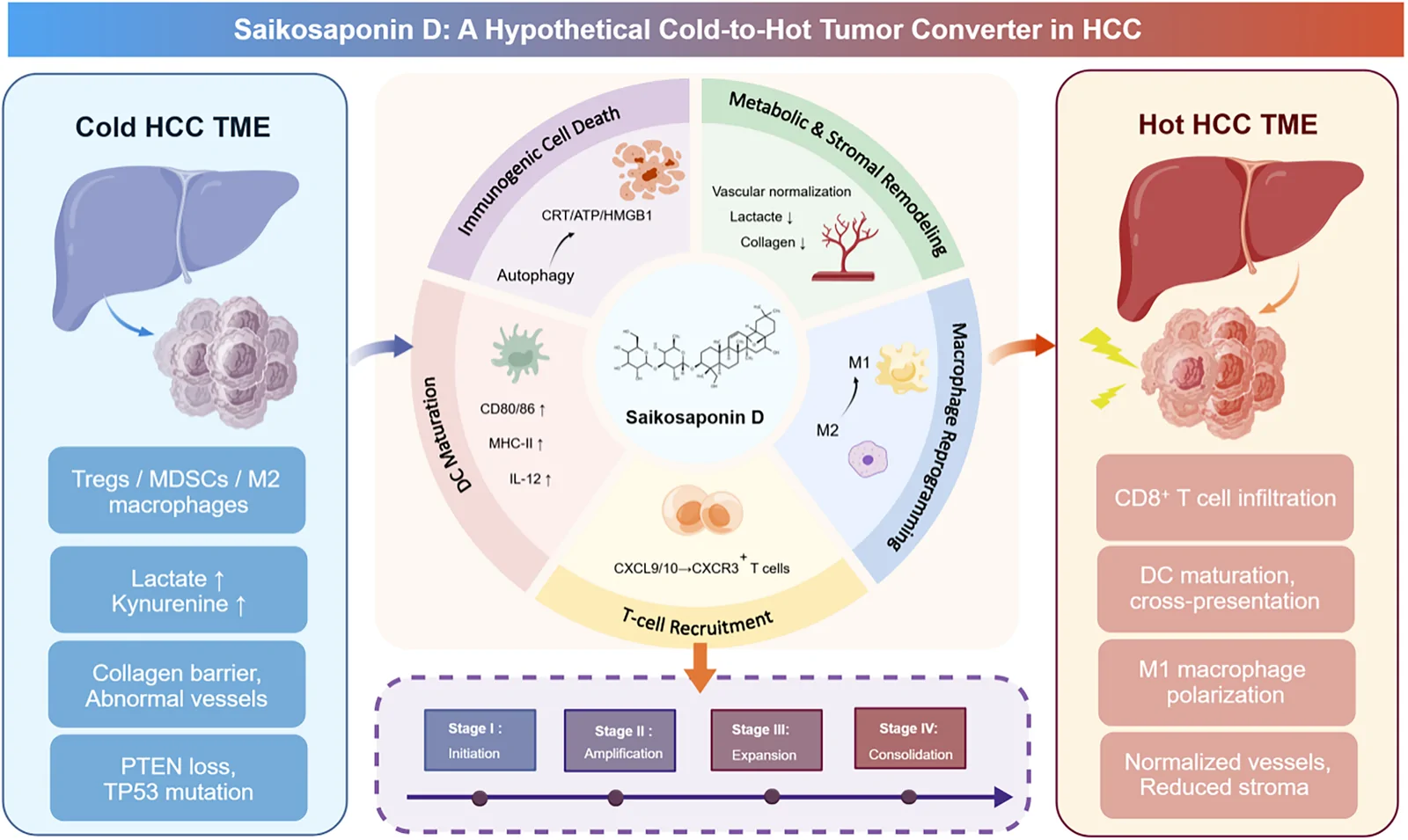

## 1 Introduction

HCC poses a significant global health challenge, ranking as the sixth most common cancer and the third leading cause of cancer-related deaths (1, 2). While there are curative options available for early-stage disease, most patients present with intermediate or advanced HCC, which necessitates systemic therapy (3). The treatment landscape has significantly evolved with the introduction of immunotherapy. Immune checkpoint inhibitors (ICIs), particularly in combination regimens such as atezolizumab plus bevacizumab and tremelimumab plus durvalumab, have established themselves as first-line standards, offering improved survival outcomes (4, 5). However, a substantial unmet need remains, as a significant proportion of patients display primary resistance, with objective response rates for single agents remaining below 20%, and many patients failing even combination therapies (6–8).

This resistance is largely driven by the TME, whose hallmark is the “cold” immune phenotype (9, 10). This “cold” state is markedly different from that of immunologically “hot” tumors. Hot tumors are characterized by abundant CD8^+^ T-cell infiltration, and a high interferon-gamma signature. In contrast, the “cold” HCC TME exhibits three main features. First, its cellular composition is predominantly composed of immunosuppressive cells such as regulatory T cells (Tregs), myeloid-derived suppressor cells (MDSCs), and M2 tumor-associated macrophages (TAMs). Second, the molecular landscape is abundant in inhibitory cytokines and metabolites. Third, physical barriers, including aberrant blood vessels and dense stroma, prevent cytotoxic T lymphocytes (CTLs) from infiltrating the tumor. Collectively, these factors create an immune desert or immune-excluded phenotype, hindering effective antitumor immunity and contributing to resistance to immune checkpoint inhibitors (ICIs) and poor patient prognosis. Therefore, transforming “cold” HCC into an immunoresponsive “hot” tumor represents a critical therapeutic objective (11). This transition is depicted schematically in **Figure 1**.

**Figure 1:**
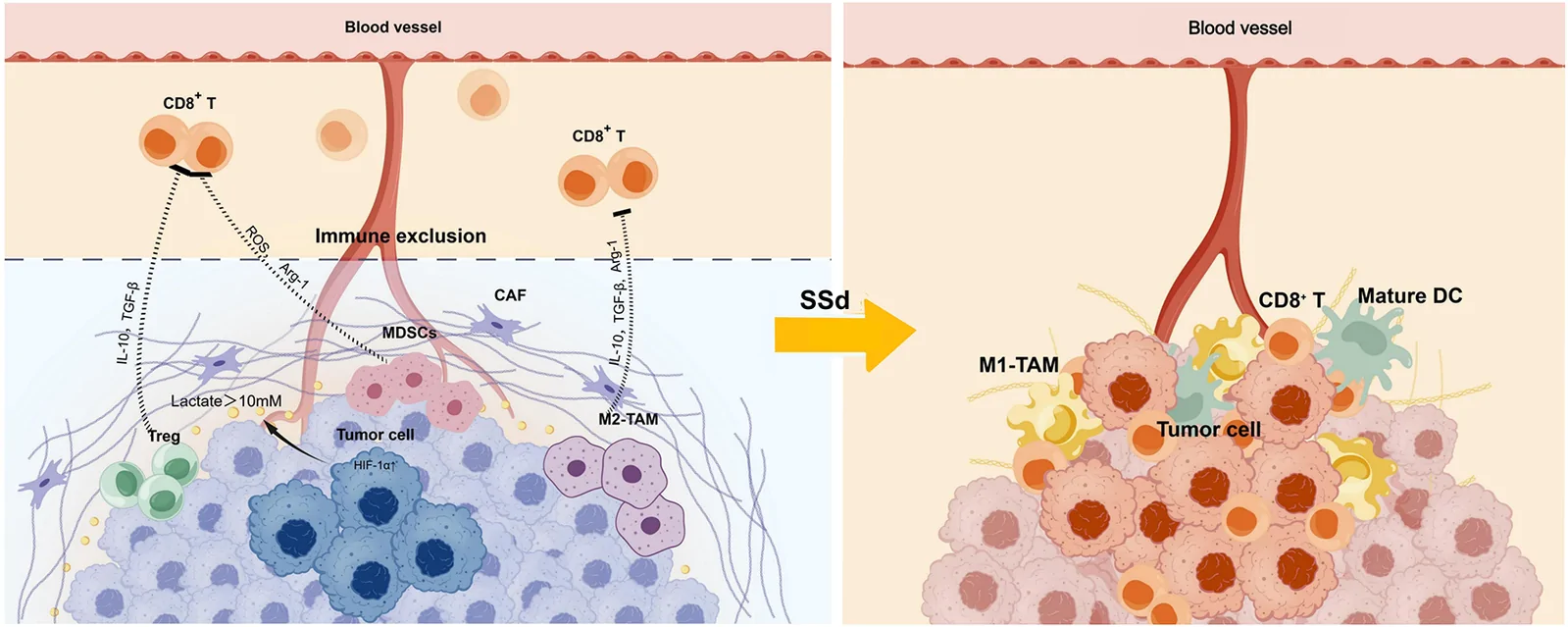
Schematic of the immunosuppressive hepatocellular carcinoma microenvironment. The left panel depicts established features of the immunosuppressive TME, including M2-TAMs, Tregs, and CAFs, alongside elevated lactate levels (>10 mM). These components collectively promote immune exclusion and limit CD8^+^ T-cell infiltration into the tumor parenchyma.The right panel illustrates proposed features of the hot TME following SSd intervention, including CD8+ T-cell infiltration, dendritic cell maturation, M1-like macrophage polarization, and normalized vasculature with reduced stroma. The central arrow denotes the hypothesized cold-to-hot conversion.

To address this challenge, strategies aimed at remodeling the immune TME have become the focus of extensive research. Naturally derived compounds possess inherently multi-target regulatory profiles and favorable safety profiles, which provide a promising avenue for therapeutic development. SSd, a triterpenoid saponin extracted from Bupleurum falcatum L., exemplifies this potential. In addition to its direct cytotoxic effects, emerging evidence identifies SSd as a multifaceted immunomodulator. SSd can influence multiple stages of the cancer-immunity cycle. For instance, it has been hypothesized to initiate immune responses through mechanisms associated with immunogenic cell death (ICD) induction, including the release of antigens and damage-associated molecular patterns (DAMPs) in preclinical non-HCC models.Direct validation of bona fide ICD in HCC remains pending.Furthermore, SSd has been shown to promote dendritic cell maturation in non-HCC *in vitro* systems,raising the hypothesis that it may enhance antigen presentation capacity within the HCC microenvironment.Additionally, in a murine pancreatic cancer model, SSd mitigates immunosuppression by inhibiting the polarization of TAMs (12) and modulating suppressive immune networks (13, 14).

However, a systematic evaluation of the role of SSd in HCC immunotherapy is currently lacking. Several critical questions remain unanswered, including its precise molecular targets, temporal regulation within the TME, and *in vivo* pharmacokinetics. The optimal strategy for synergizing SSd with existing immunotherapies, including the appropriate dosing sequence and its mechanisms of action in conjunction with ICIs, is also unclear. This review presents a hypothesis-driven framework to explore whether SSd can convert “cold” HCC into “hot” tumors. Unlike prior catalogues of SSd’s isolated antitumor effects, this framework integrates them within a cold-to-hot immunological timeline and a four-stage temporal model. We explicitly acknowledge that this framework is speculative and intended to guide future investigations, rather than to establish validated therapeutic principles. We will outline the immunomodulatory mechanisms, evaluate combination strategies, and discuss challenges and future directions for clinical translation.

## 2 Materials and methods

### 2.1 Literature search strategy

This review is a hypothesis-generating narrative mechanistic synthesis of preclinical evidence. It was not registered in PROSPERO because intervention-based systematic review criteria do not apply. PRISMA 2020 reporting was followed only where applicable to this narrative synthesis. We systematically searched PubMed, EMBASE, Web of Science, and Cochrane Library from January 1, 2020 to December 31, 2025, using combinations of the following keywords: “Saikosaponin D”, “SSd”, “hepatocellular carcinoma”, “solid tumors”, “tumor immune”, “immunomodulation”, “immune checkpoint inhibitors”, and “immunogenic cell death”. Given the limited number of SSd-specific studies in HCC, the search was broadened to include solid tumor immunology literature to inform mechanistic inferences. Reference lists of retrieved articles were also manually screened. The exact search terms and their Boolean combinations are detailed in full in **Supplementary Table S1**, including database-specific field mappings where applicable. Duplicate removal was performed using reference-manager software (EndNote) followed by manual checks for records appearing in different languages or with abbreviated titles.

### 2.2 Inclusion and exclusion criteria

Studies were included if they: (1) investigated the immunomodulatory effects of SSd in HCC or, secondarily, in other solid tumors, with their non-HCC origin explicitly acknowledged; (2) used SSd as a monotherapy or in combination with clearly defined dosing; and (3) reported immune-related outcomes. Studies were excluded if they focused on other saikosaponins or compound formulations where SSd’s independent effect could not be isolated, involved non-solid tumors or solely reported direct cytotoxicity without immune mechanisms, or were case reports, opinion pieces, non-peer-reviewed literature, or duplicate publications without full-text availability. Quality assessment was performed independently by two reviewers using SYRCLE’s risk-of-bias tool for animal studies and a tailored checklist for *in vitro* studies, covering cell-line authentication, mycoplasma status, dose/concentration and exposure time, replicates and statistics, comparator, and defined immune readouts.

### 2.3 Search results and study selection

Two investigators independently screened titles and abstracts, followed by full-text evaluation of potentially relevant records. Disagreements were resolved by discussion or by consultation with a third reviewer. Reasons for full-text exclusion are summarized in PRISMA flow diagram. Data on first author, publication year, model system, SSd dosing regimen, key findings, and study quality indicators were extracted using a standardized form. For non-HCC tumor models, tumor type and model system were recorded to grade evidence relevance. The initial search yielded 306 records, from which 139 publications were ultimately included to inform the mechanistic framework. Of these, 29 are primary experimental studies with direct SSd-related data; the remaining 110 provide supporting context on HCC immunobiology. Given the predominance of indirect evidence, this review is structured as a hypothesis-generating framework, with all cross-tumor inferences explicitly identified throughout the text. The screening and selection process is summarized in the PRISMA flow diagram (**Figure 2**).

**Figure 2:**
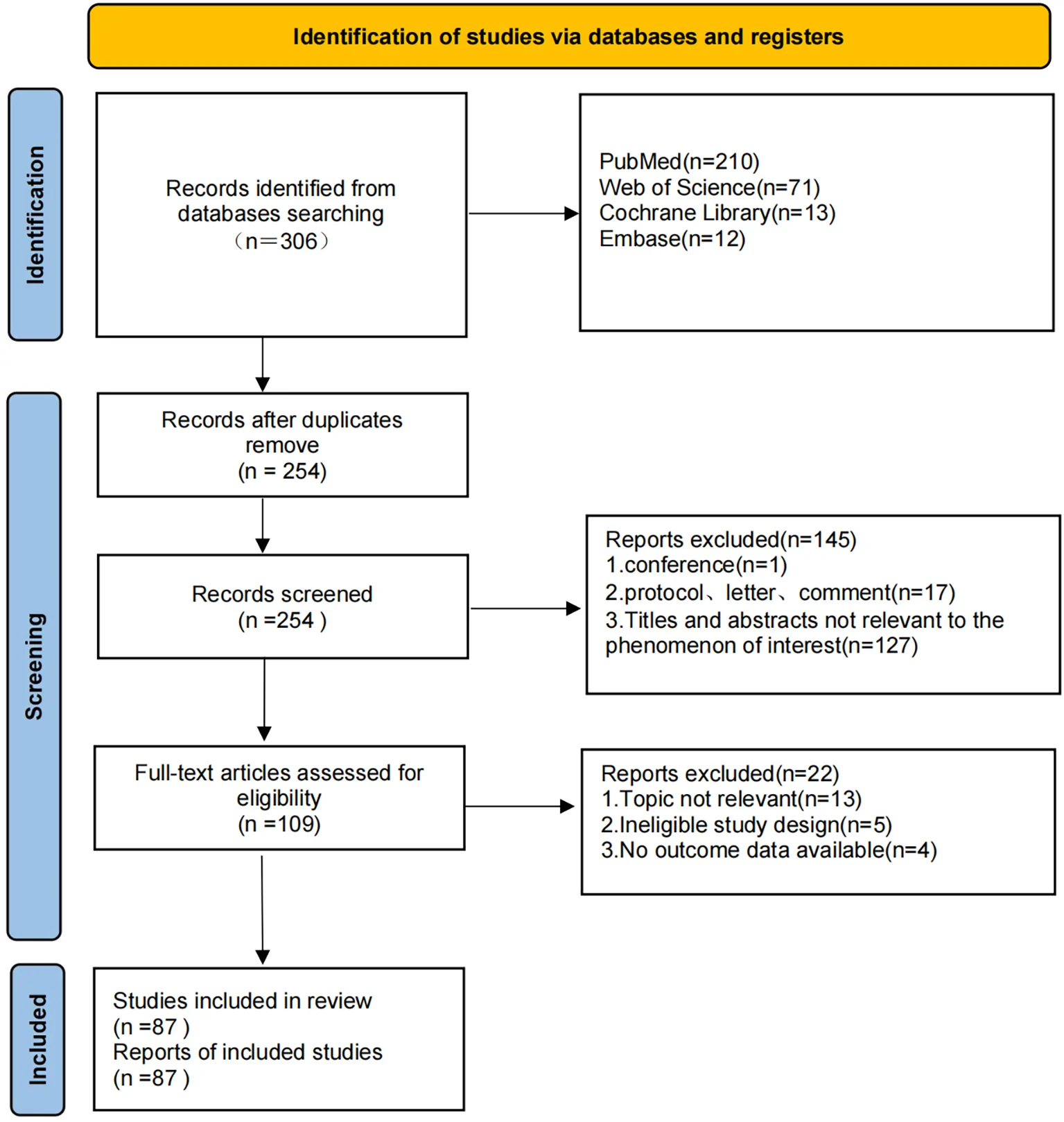
The PRISMA flow diagram summarizes studies identified through database searching and systematic screening (n = 87). An additional 52 studies were included through manual reference list screening and citation tracking, bringing the total number of references cited in the review to 139.

## 3 Chemical structure, pharmacokinetics, and general antitumor properties of SSd

### 3.1 Chemical structure of SSD

SSd is an epoxy ether-type pentacyclic triterpenoid saponin isolated from the roots of Bupleurum spp., with the molecular formula C_42_H_68_O_13_. It falls under the category of oleanane-type triterpenoid saponins (15). The aglycone of SSd is saikogenin D, the non-sugar portion of the molecule. Saikogenin D contains a 13β,28α-epoxy bridge between C-13 and C-28. This bridge locks the aglycone into a rigid three-dimensional conformation. The rigid conformation favors binding to biological targets and is associated with preserved immunomodulatory activity. Opening the epoxy bridge yields saikosaponin b_2_, which lacks this rigid conformation and shows markedly diminished activity (16). In existing animal experimental studies, SSd has primarily been administered through gastric lavage and intraperitoneal injection.

### 3.2 Pharmacological effects of SSD

SSd demonstrates a broad range of pharmacological activities, including anti-inflammatory, antioxidant, anti-apoptotic, anti-fibrotic, immunomodulatory, and antitumor effects. In lipopolysaccharide (LPS)-stimulated RAW264.7 macrophages, SSd suppresses NF-κB activation and reduces the expression of inducible nitric oxide synthase (iNOS), cyclooxygenase-2 (COX-2), tumor necrosis factor-alpha (TNF-α), and interleukin-6 (IL-6) (17). These findings are consistent with SSd’s potential to modulate inflammation in the context of chronic liver disease.

Beyond its anti-inflammatory activity, SSd displays complex immunomodulatory effects that vary by cellular context. In terms of its antitumor effects, SSd inhibits HCC progression through multiple mechanisms. It downregulates the HIF-1α (18) and p-STAT3/C/EBPβ (19) signalling pathways, which inhibits COX-2 expression and the synthesis of prostaglandin E2, thereby suppressing HCC cell proliferation and inducing apoptosis. Furthermore, SSd enhances the radiosensitivity of HCC cells by modulating the G0/G1 and G2/M cell cycle checkpoints (20). It upregulates the expression of p53 and Bax while downregulating Bcl-2 to promote apoptosis. Under hypoxic conditions, SSd further enhances radiosensitivity by inhibiting HIF-1α.

In summary, the aforementioned advancements in pharmacological research highlight the mechanistic versatility of SSd in preclinical settings. However, as detailed in the following section, the translation of these *in vitro* and non-orthotopic findings into clinically relevant outcomes confronts formidable pharmacokinetic barriers.

### 3.3 Potential toxicity and safety constraints

Saikosaponin D is a major toxic constituent of Bupleuri Radix, and its hepatotoxicity, hemolysis, and cardiotoxicity are the main safety concerns limiting its therapeutic development (21).

Hepatotoxicity is the best characterized. In human L-O2 hepatocytes, SSd induces Fas-mediated apoptosis. In mice, one week of treatment at 300 mg/kg causes massive hepatocyte apoptosis and histological liver injury (22). Because HCC often develops in chronically diseased livers, this hepatotoxic potential is particularly relevant to therapeutic development. Hemolysis is intrinsic to the saponin structure. SSd interacts with erythrocyte membranes and causes dose-dependent lysis at 1.0–5.0 μg/mL (23). This range overlaps with that required for its immunomodulatory effects *in vitro*. As a SERCA inhibitor, SSd disrupts cardiomyocyte calcium homeostasis and is more toxic than other saikosaponins at equivalent concentrations (24).

These toxicities make hepatocyte-targeted nano-delivery a safety-enabling strategy, not merely a means to improve bioavailability.

### 3.4 Pharmacokinetic profile and translational bottlenecks

SSd faces a major pharmacokinetic obstacle: the concentrations required for its *in vitro* immune effects cannot be achieved *in vivo*. This is largely due to its classification as an oleanane-type triterpenoid saponin, which exhibits poor gastrointestinal permeability because of its hydrophilic glycosidic groups. In studies using Caco-2 cell and rat intestinal perfusion models, its apparent permeability coefficient indicates negligible passive absorption, while P-glycoprotein efflux further restricts its uptake (25).

Pharmacokinetic studies of the structurally related C-23 epimer, saikosaponin A highlight the severity of this issue: over a dose range of 50–200 mg/kg, its oral bioavailability is approximately 0.04% (26). As a structural epimer of SSd, SSa shares similar physicochemical properties, underscores the pharmacokinetic barriers that SSd itself is likely to face. In gastric fluid, the characteristic 13β,28α-epoxy bridge of SSd undergoes acid-catalysed ring opening within 30 minutes. Subsequently, intestinal β-glucosidases mediate stepwise deglycosylation, resulting in prosaikogenin D and saikogenin D. Hepatic CYP450 enzymes further oxidise these intermediates. Whether the immunomodulatory effects observed *in vitro* are mediated by SSd itself or by its metabolites remains undefined.

The *in vitro* immunostimulatory concentrations of SSd are substantially higher than those achievable *in vivo* through oral administration. For DC maturation, the effective concentration ranges from 5–20 μM. For macrophage repolarization, the required range is 1 to 5 μM. The oral bioavailability of saikosaponin A, a relevant proxy, is extremely low, with a Cmax of approximately 0.01 μg/mL.

Advanced delivery systems present a promising solution. Macrophage membrane-coated PLGA nanoparticles incorporating T7 peptide targeting have been shown to enhance tumor-specific accumulation of SSd and reduce systemic toxicity (27). Hepatocyte-targeted liposomal formulations, such as galactosylated liposomes, represent additional strategies to improve intratumoral drug exposure. Whether these carriers can achieve the necessary concentrations for immune activation remains to be determined.

In summary, the exceedingly low oral bioavailability and significant presystemic metabolism of SSd constitute the principal translational barriers to its immunomodulatory application in HCC (28). The mechanistic framework presented herein should be viewed as a hypothesis-generating construct, the validity of which relies on pharmacokinetic validation in orthotopic tumor models.

## 4 The “cold” HCC immune microenvironment: phenotype and mechanisms

HCC frequently presents as an immunologically “cold” tumor, with the immune-excluded phenotype predominating, characterized by limited CTLs infiltration and a superimposed immunosuppressive network (29). Inhibitory cell populations, metabolic barriers, and tumor-intrinsic signaling collectively sustain this state. SSd targets multiple nodes within these barriers, providing a mechanistic basis for cold-to-hot conversion (**Figure 3**).

**Figure 3:**
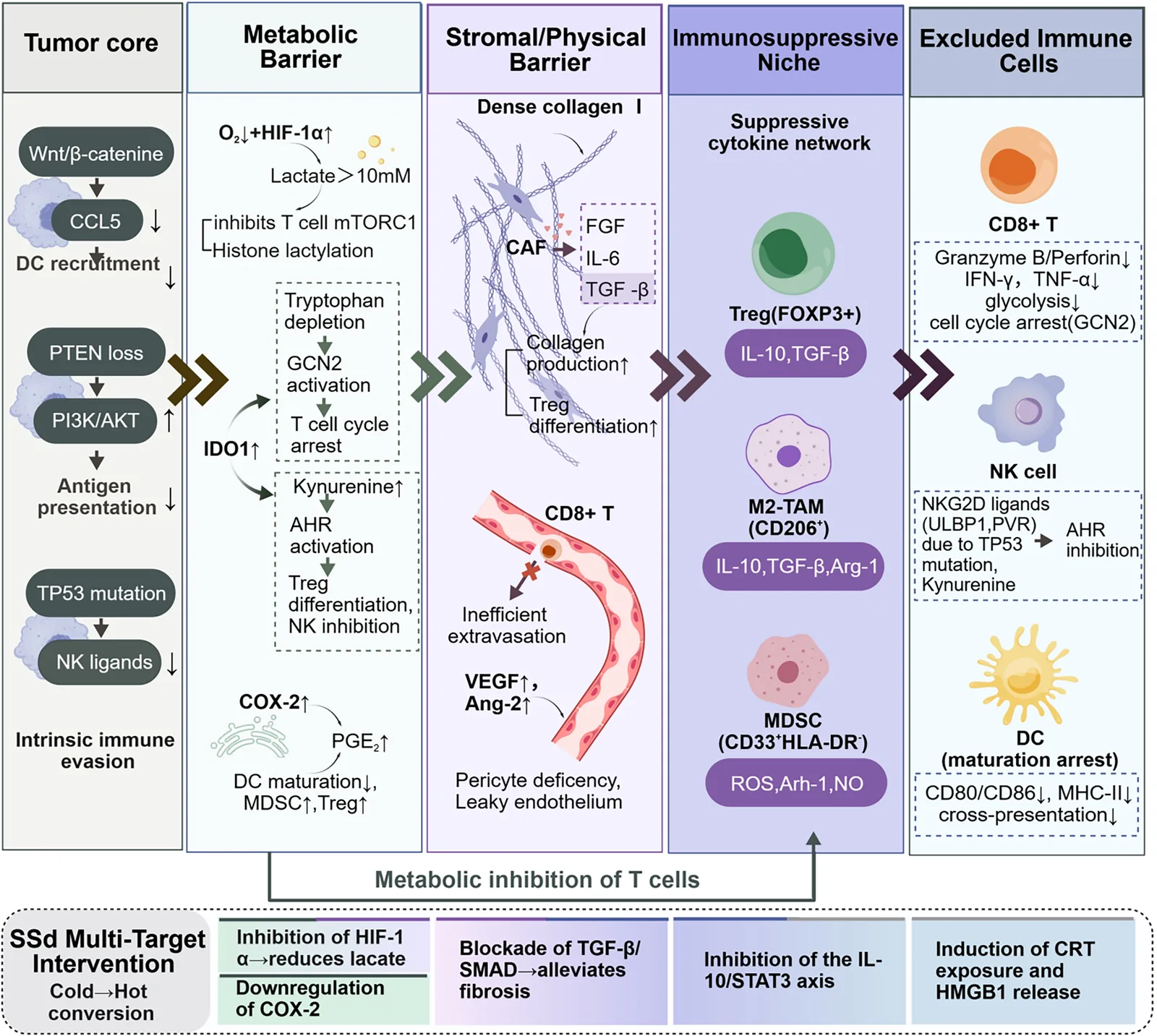
Schematic overview of the “cold” hepatocellular carcinoma microenvironment, highlighting tumor-intrinsic genetic alterations, metabolic and stromal barriers, immunosuppressive cell populations, and the multi-target intervention points of SSd. The colored lines above each SSd intervention indicate the corresponding “cold” tumor modules it targets; multiple colors denote action on more than one module.

### 4.1 Immunosuppressive cells and cytokine axis

The term “cold” tumor microenvironment comprises immune-desert, immune-excluded, and immunosuppressed (30, 31). In HCC, the immunosuppressed subphenotype predominates, with infiltration of regulatory T cells, myeloid-derived suppressor cells, and M2-polarized tumor-associated macrophages, along with aberrant secretion of inhibitory cytokines (32). This network suppresses antitumor immunity and contributes to resistance to immune checkpoint blockade.

Dendritic cell dysfunction exemplifies this immunosuppressive network. Tumor-infiltrating DCs in HCC frequently display maturation arrest and impaired antigen presentation, hindering effective priming of tumor-specific CTLs. The IL-10/STAT3 and TGF-β/SMAD axes are established suppressors of DC maturation and CTL effector function (33, 34). Hepatic DC subsets (35–37) and single-cell transcriptomic profiling (38, 39) have further revealed widespread dysfunction and exhaustion of DCs and T cells in HCC. SSd inhibits STAT3 and TGF-β signaling in preclinical models, suggesting it may restore DC competence and CTL cytotoxicity in the HCC microenvironment (**Figure 3**). Direct validation in HCC-infiltrating DCs remains lacking.

### 4.2 Metabolic reprogramming and physical barriers

HCC creates metabolic and physical barriers that prevent CTL infiltration and function. Hypoxia-induced glycolysis, amino acid depletion, and stromal stiffening constitute a self-reinforcing network that sustains the “cold” immune phenotype. SSd can disrupt this network by targeting each of these barriers individually.

#### 4.2.1 Hypoxia-glycolysis-lactate axis

Rapid tumor proliferation stabilizes HIF-1α and driving aerobic glycolysis, the Warburg effect, which leads to the accumulation of lactate (40, 41). In HCC, intratumoral lactate levels often exceed 10–20 mM and are correlated with PD-L1 expression and resistance to anti-PD-1 therapy. Lactatesuppresses mTORC1 signalling and effector cytokine production in CTLs (42), alters the α-ketoglutarate/2-hydroxyglutarate balance (43) and promotes histone lactylation that reinforces HIF-1α-driven immunosuppressive (44, 45). It also suppresses T cell and NK cell function, induction of regulatory T cells, and M2 polarization of tumor-associated macrophages (46).

SSd inhibits HIF-1α in hepatoma cells, which may reduce lactate accumulation and relieve T-cell metabolic suppression (**Figure 3**). HIF-1α itself stabilized by lactylation, creating a feed-forward circuit that sustains glycolysis-driven immunosuppression (47). By suppressing HIF-1α, SSd may disrupt the positive feedback loop between glycolysis and lactate production (48). T-cell metabolic suppression in HCC can also be driven by IDO1-mediated tryptophan depletion (49), and SSd’s inhibition of STAT3 may reduce IDO1 expression. Metabolic stress activates AMPK and inhibits mTOR, thereby inducing autophagy. SSd activates the CaMKKβ-AMPK-mTOR pathway and induces autophagy in autosomal dominant polycystic kidney disease (ADPKD) cells (50), suggesting that SSd may also modulate autophagy in HCC as part of its metabolic effects.

#### 4.2.2 Tryptophan-kynurenine pathway

Indoleamine 2,3-dioxygenase 1 (IDO1) converts tryptophan to kynurenine (Kyn), resulting in the generation of potent immunosuppressive metabolites that are often upregulated in HCC tumor cells and infiltrating dendritic cells (51). Tryptophan depletion activates GCN2, leading to CTL cycle arrest and anergy. Meanwhile, Kyn interacts with the aryl hydrocarbon receptor (AHR), promoting the differentiation of regulatory Treg and impairing the cytotoxicity of NK cells (52, 53). These pathways demonstrate a synergistic “dual sensitization”: the activation of GCN2 enhances the expression of the Kyn transporter LAT1, increasing T cell sensitivity to residual Kyn (54).

In HCC, increased expression of IDO1 is associated with a poor response to immune checkpoint inhibitors and a reduced overall survival (55). The inhibition of IDO1 in combination with anti-PD-1 therapy has progressed through phase I/II and phase III clinical trials, validating this metabolic checkpoint as a targetable pathway, with ongoing efforts to optimize therapeutic strategies (56, 57).

SSd inhibits the phosphorylation of STAT3 in hepatoma cells. Because STAT3 activation has been shown to upregulate IDO1 transcription in several tumor contexts (58), it is plausible that SSd may indirectly mitigate Kyn-mediated immunosuppression(**Figure 3**). This STAT3-IDO1 axis presents a promising mechanism for SSd-mediated remodeling of the “cold” microenvironment.

#### 4.2.3 Vascular and stromal barriers

VEGF-driven aberrant angiogenesis produces structurally abnormal tumor vasculature characterised by inadequate pericyte coverage and disrupted expression of endothelial adhesion molecules. This paradoxically hinders T cell transendothelial migration despite increased vascular permeability.

Beyond their structural role, cancer-associated fibroblasts (CAFs) can be activated by tumor-derived lactate through a NAD^+^/p62-dependent mechanism, which reinforces the desmoplastic and immunosuppressive microenvironment (59). Concurrently, CAFs synthesise type I collagen (collagen-I) and crosslinked extracellular matrix, creating a desmoplastic stroma. In HCC, FAP+ CAFs are associated with reduced CTL infiltration, as they recruit regulatory T cells and M2 macrophages while inhibiting CD8+ T cell and NK cell infiltration through cytokine secretion (60). The deposition of collagen-I is associated with the exclusion of CD8+ T cells and independently predicts poor prognosis in resected HCC (61).

SSd downregulates TGF-β1 signalling and reduces the expression of fibrotic markers, such as collagen-I, in hepatic stellate cells (62). Suppression of TGF-β-mediated fibrogenesis by SSd reduces collagen deposition and physical barrier formation, facilitating CTL infiltration into the tumor parenchyma (**Figure 3**).

### 4.3 Cancer cell-intrinsic mechanisms of immune evasion

Beyond the cellular and metabolic barriers imposed by the microenvironment, HCC cells also employ intrinsic genetic programs to evade immune detection and elimination. Activating mutations in CTNNB1 (which encodes β-catenin) repress of chemokines such as CCL5, which are crucial for the recruitment of CD103+ DCs into the tumor microenvironment (63). This impairment in DC recruitment hinders antigen presentation and subsequent activation of CD8+ T cells, leading to an immune-excluded phenotype that is resistant to immune checkpoint inhibitor therapy (64). Whether SSd directly modulates β-catenin signaling in HCC is unknown. One possibility is that SSd may partially compensate for defective DC recruitment through its broader effects on antigen-presenting cells (**Figure 3**).

PTEN lossactivates PI3K-AKT signalling pathway, which is associated with immune dysregulation and increased resistance to T cell-mediated immunotherapy in HCC (65). This mechanism is particularly relevant to SSd because PI3K/AKT/mTOR is one of its established targets (**Figure 3**). If SSd inhibits this pathway in HCC, it could potentially reverse the immunosuppressive consequences of PTEN loss, converting an immune-deficient microenvironment into one permissive for T cell priming.

TP53 mutation downregulate NKG2D and DNAM1 ligands, reducing NK cell recognition of tumor cells (66). SSd-induced immunogenic cell death and CRT exposure (**Figure 3**) could enhance NK cell activation through mechanisms independent of NKG2D ligand expression, but this has not been examined.

In summary, these cancer-cell-intrinsic mechanisms converge to create an immunologically “cold” tumor microenvironment in HCC. Wnt/β-catenin activation, PTEN loss, and TP53 mutations represent distinct yet complementary pathways that collectively compromise antigen presentation, T-cell priming, and innate immune recognition. These genetic alterations frequently coexist in HCC, suggesting a collaborative immune evasion strategy that renders tumors resistant to monotherapy approaches. Understanding these intrinsic mechanisms is therefore essential for developing rational combination strategies that target both tumor cells and the immune microenvironment—potentially relieving immune exclusion and overcoming the “cold” phenotype.

## 5 Preclinical evidence for SSd-mediated remodeling

A condensed summary of the 29 primary studies providing direct SSd-related data is presented in **Table 1**, categorized by evidence grade (D-HCC, I-nonHCC, or I-IV). The complete evidence matrix, cataloguing each study by model system, dose, immune-related findings, pathway, and key limitation, is provided in **Supplementary Table S2**.

**Table 1:** Summary of primary SSd-related studies by evidence grade.

Evidence grade	Definition	Number of studies	Representative references
D-HCC	Direct HCC/hepatocellular evidence	11	(13, 14, 18–20, 61, 69, 99, 110, 121)
I-nonHCC	Indirect, non-HCC tumors	10	(12, 22, 86–88, 105, 111–113, 127, 128)
I-IV	*In vitro*/non-tumor mechanistic	8	(23–26, 50, 75, 98, 129)

### 5.1 Antigen release: mechanistic basis for immunogenic cell death induction

ICD is a key mechanism that links tumor cell stress to adaptive immunity. It serves as the initial trigger for converting immunologically “cold” tumors into “hot” phenotypes. It is operationally defined by the spatiotemporally coordinated release of DAMPs. These include CRT exposure on the cell surface, active ATP secretion, and passive HMGB1 release. Collectively, these signals provide “eat me,” “find me,” and “danger” cues for the recruitment and activation of dendritic cells.

Direct evidence for SSd-induced ICD in HCC is lacking. Existing studies have not utilised the gold-standard assays for ICD certification, which include flow cytometric quantification of CRT surface exposure, luminometric measurement of extracellular ATP, or vaccination experiments demonstrating protective immunity against live tumor cell challenges (67). This is partly attributable to the complex aetiology of HCC, which includes chronic hepatitis B or C infection, alcohol-induced liver disease, and non-alcoholic steatohepatitis. Each of these conditions generates its own DAMPs, potentially confounding the detection of tumor-specific ICD signals (68). These factors underscore the need for systematic ICD validation in HCC models with well-defined aetiologies.

Indirect evidence from studies using HCC cell lines suggests that SSd creates cellular conditions that are conducive to the execution of ICD. In HepG2 and SMMC-7721 hepatoma cells, SSd induces autophagy by inhibiting the PI3K/AKT/mTOR pathway (69). Hypoxia, a hallmark of the HCC microenvironment, is known to influence autophagic responses and ICD outcomes through HIF-1α stabilization (70). Autophagy acts as a critical auxiliary mechanism for ICD, promoting the lysosomal-dependent translocation of CRT to the cell surface and the non-lytic release of HMGB1 from the nucleus to the extracellular space (71, 72). The temporal dynamics of this relationship remain undefined; whether SSd-induced autophagy precedes, accompanies, or follows the execution of cell death will determine its relevance to bona fide ICD as opposed to collateral stress responses.

SSd-mediated HIF-1α inhibition has implications for ICD. SSd effectively inhibits HIF-1α in hepatoma cells under hypoxic conditions through the p-STAT3 pathway (73). By suppressing HIF-1α, SSd may alleviate hypoxia-driven immunosuppression in HCC, sensitizing tumor cells to immune recognition.

Extrapolating ICD mechanisms from other tumor types to HCC requires caution. The chronically inflamed liver microenvironment is characterised by TLR4 activation and sustained inflammation, which may alter immune responsiveness to danger signals (74). Furthermore, the hepatitis B surface antigen (HBsAg) and hepatitis C core antigen, which are abundantly present in virally induced HCC, could act as decoy antigens that compete with tumor neoantigens for immune recognition during ICD-induced responses.

### 5.2 Effector recruitment and empowerment: enhancing CD8^+^ T cell infiltration and cytotoxicity

Dendritic cells serve as a bridge linking innate pathogen recognition to adaptive antitumor immunity. In solid malignancies such as hepatocellular carcinoma, tumor-infiltrating dendritic cells display phenotypic and functional paralysis, characterised by reduced co-stimulatory molecule expression, diminished IL-12 production, and impaired priming of naïve CD8^+^ T cells. The hepatic microenvironment amplifies these dysfunctions. Restoring dendritic cell competence is essential for transforming “cold” HCC into immunologically responsive “hot” tumors.

In human monocyte-derived DCs (mo-DCs), SSd upregulates CD80, CD86, and MHC-II on the cell surface. It also enhances the secretion of bioactive IL-12p70. Functionally, SSd-matured DCs exhibit an increased capacity to stimulate allogeneic T-cell proliferation and show elevated IL-12 secretion, which drives T helper 1 (Th1) polarization and interferon-γ production. Whether SSd can promote Th1 polarization or IFN-γ production within the HCC microenvironment, however, remains to be determined. At concentrations of 5–20 μM, SSd upregulates CD40, CD83, CD80, CD86 and promotes IL-12 secretion in human monocyte-derived DCs, consistent with the phenotypic and functional requirements for DC-mediated T-cell priming (75). This integrated maturation profile contrasts with partial activation states that may induce T-cell anergy or tolerance, thereby providing SSd with a mechanistic advantage in overcoming the dysfunctional antigen presentation characteristic of the HCC microenvironment (76).

In the HCC microenvironment, tumor-infiltrating dendritic cells (tiDCs) face several layers of suppressive regulation. Hypoxia-induced stabilization of HIF-1α hinders dendritic cell maturation and the antigen-processing machinery (77, 78). Accumulated lactate inhibits dendritic cell glycolytic metabolism and IL-12 production through histone lactylation-dependent epigenetic reprogramming (79, 80). Elevated levels of TGF-β and IL-10 activate STAT3 and SMAD signalling pathways, reinforcing immune tolerance (81, 82).

Cross-presentation is the processing of exogenous antigens onto MHC-I for CD8^+^ T cell activation, and it is a key function of DCs in antitumor immunity. SSd enhances cross-presentation-related markers in monocyte-derived DCs, but direct evidence for cross-presentation of HCC antigens remains absent. This gap matters in HCC, where β-catenin activation and an immunosuppressive microenvironment often impair antigen presentation (83, 84). Addressing this question will be important for evaluating the potential of SSd to support CD8^+^ T cell responses in HCC.

### 5.3 Effector cell recruitment and functional reprogramming in the hepatic microenvironment

Hepatocellular carcinoma poses a significant obstacle to T-cell-mediated immunity. Effector T cells are often scarce, actively excluded from the tumor parenchyma, or functionally suppressed within the immunosuppressive environment. SSd tackles this dual challenge through complementary mechanisms that utilise both direct chemokine induction and metabolic reprogramming, providing distinct advantages over single-target interventions.

CXCR3 ligand induction and hepatic T-cell trafficking. The liver’s unique vascular architecture creates both opportunities and obstacles for effector T-cell recruitment. The fenestrated sinusoidal endothelium, slow blood flow, and constitutive intrahepatic lymphocyte surveillance collectively shape this dual role. While circulating activated T cells continuously survey the hepatic vasculature, efficient extravasation into HCC nodules requires active chemokine gradients that overcome the sinusoidal barrier. SSd promotes Th1 polarization and IFN-γ production through mTOR-dependent pathways (85), a property shared with its epimer saikosaponin A in breast cancer models (86). In HCC, intratumoral CXCL9 density correlates with CD8^+^ T-cell infiltration, granzyme B expression, and objective response to immune checkpoint inhibitors. SSd’s impact on T-cell recruitment extends beyond chemokine provision. It suppresses hepatic stellate cell activation and collagen-I deposition (87, 88), and may also promote vascular normalization by inhibiting HIF-1α and VEGF signaling (89). These effects may alleviate the desmoplastic stromal barrier in advanced HCC, creating conditions that favor T-cell penetration into the tumor parenchyma.

Vascular normalization opens the gate for immune entry. Beyond chemokine gradients and stromal remodeling, the physical structure of tumor vasculature critically determines whether circulating T cells can successfully extravasate into the tumor parenchyma. In HCC, tumor vasculature is characteristically disorganized, with poor pericyte coverage, reduced expression of endothelial adhesion molecules, and tortuous vessels. These structural abnormalities prevent circulating T cells from effectively arresting on the endothelium and extravasating into the tumor tissue. SSd suppresses HIF-1α and downstream VEGF-driven angiogenic signaling, which may promote vascular normalization (90), including restored pericyte coverage and re-expresses adhesion of molecules such as ICAM-1 and VCAM-1, that are essential for T-cell transmigration. This remodeling creates functional gateways within localized tumor regions (91), allowing circulating immune effectors to efficiently interact with the endothelium, stably arrest under hemodynamic shear stress, and extravasate into the tumor parenchyma (92, 93).

Metabolic empowerment of intrahepatic T-cell function. Once recruited, T cells encounter the metabolically hostile HCC microenvironment: hypoxia, lactate accumulation, and tryptophan depletion collectively suppress mTOR signaling, mitochondrial oxidative phosphorylation, and effector cytokine production (94, 95). The tryptophan–kynurenine pathway is sustained by multiple signalling inputs, including Hedgehog/GLI, JAK/STAT (96) and Wnt/β-catenin (97) pathways. By inhibiting STAT3 phosphorylation, SSd may reduce IDO1 expression and thereby relieve GCN2-dependent metabolic constraints in T cells. The broader STAT3–IDO1/COX-2/HIF-1α axis and its downstream metabolites are discussed above. In parallel, SSd induces autophagy in hepatoma cells through the CaMKKβ-AMPK-mTOR axis (98, 99). This pathway is initiated by SERCA inhibition and may contribute to immunogenic cell death, thereby providing metabolic substrates for sustained T-cell activation (100).

Functional enhancement of cytotoxic capacity. SSd augments effector T-cell function through metabolic and microenvironmental modulation. By suppressing HIF-1α stabilization, SSd mitigates hypoxia-driven metabolic suppression and lactate accumulation, thereby relieving mTORC1 inhibition and restoring T-cell glycolytic capacity. SSd-induced autophagy provides metabolic substrates for sustained T-cell activation while promoting immunogenic cell death. Additionally, IDO1-driven tryptophan catabolism activates GCN2 and impairs effector T-cell function (101), a pathway that SSd may indirectly attenuate through STAT3 inhibition. These coordinated effects position SSd to overcome T-cell exclusion and exhaustion in HCC immunotherapy.

### 5.4 Reversal of immunosuppressive networks: macrophage-centric remodeling

The HCC immune microenvironment is dominated by myeloid-derived suppressive networks that actively subvert antitumor immunity. SSd targets these networks with remarkable specificity, reprogramming macrophage phenotypes and disrupting metabolic suppression mechanisms that are particularly prominent in hepatic malignancy.

TAMs in HCC are predominantly M2-polarized. They express CD206, IL-10, and TGF-β, which suppress T-cell activation and promote Treg expansion. In a murine pancreatic cancer model, SSd deactivates PI3K/AKT/mTOR and STAT6 signaling in tumor-associated macrophages. This blocks M2 polarization and promotes M1-like macrophage accumulation and pro-inflammatory cytokine production, which is expected to increase CXCL9/10 and IL-12/IFN-γ release and promote CD8^+^ T-cell infiltration. By analogy, we hypothesize that SSd could favor M1-like repolarization of HCC-associated Kupffer cells and TAMs, but direct HCC evidence is currently lacking. Arginase-1 released by myeloid cells is known to suppress T-cell responses (102). This M2-to-M1 shift has functional consequences: reduced Arg-1 activity restores L-arginine availability for T-cell signaling (103), while iNOS-derived nitric oxide enhances macrophage cytotoxicity against tumor cells (104). Notably, SSd also promotes M2 polarization in autoimmune disease models, illustrating its context-dependent immunomodulatory capacity (105). In the tumor microenvironment, EGFR/PI3K/AKT/mTOR-driven signals promote M2 polarization (106). SSd acts against these signals and favors M1-like repolarization. This effect is amplified in the liver, where Kupffer cells and infiltrating macrophages are key drivers of immune tolerance. Reversing their phenotype could disrupt the self-sustaining suppressive circuits that sustain “cold” tumors. The multifaceted roles of TAMs in HCC pathogenesis, prognosis, and therapy resistance have been reviewed (107).

SSd modulates the cytokine environment toward an effector phenotype. In HCC, TGF-β signaling promotes Treg differentiation, suppresses CTL granzyme and perforin expression, and induces mitochondrial dysfunction (108, 109). By attenuating STAT3 signaling in non-HCC models (110, 111), SSd reduces TGF-β-driven immunosuppression, which in turn limits Treg accumulation and restores cytotoxic T-cell metabolic fitness. Furthermore, SSd-mediated disruption of IL-6-driven STAT3 activation and TGF-β signaling breaks the self-sustaining suppressive circuits between tumor cells and macrophages that characterize “cold” tumors. SSd also dampens NLRP3 inflammasome activation in hepatic fibrogenesis (112), supporting its broader capacity to interrupt inflammatory circuits that sustain immunosuppressive myeloid networks. These actions position SSd as a comprehensive approach to macrophage-centric immune remodeling in HCC.

SSd inhibits STAT3 phosphorylation (113), a central node for MDSC survival and suppressive function (114, 115). In HCC, where viral proteins and tumor-derived factors drive STAT3 activation and MDSC expansion (116, 117), this inhibition may be particularly impactful.The anticipated reduction in Arg-1 activity mediated by STAT3 inhibition would mitigate the metabolic competition that MDSCs and M2 macrophages impose on T cells, restoring amino acid availability for effector cell function.

The chronic inflammatory background of viral and metabolic liver disease creates unique immunosuppressive pressures that SSd is well positioned to address.In hepatitis B-associated HCC, the viral X protein activates STAT3 and NF-κB to promote MDSC accumulation and PD-L1 expression (118); SSd’s concurrent inhibition of these pathways offers mechanistic synergy, which could be further leveraged in rational combination strategies (119). In non-alcoholic steatohepatitis-associated HCC, metabolic stress and lipotoxicity drive macrophage inflammatory protein expression and T-cell dysfunction; SSd’s metabolic reprogramming effects may counteract these obesity-related immune defects. This contextual specificity addresses both liver disease-derived and tumor-intrinsic immunosuppression.

### 5.5 Mechanistic integration: SSd as a hypothetical network-disrupting “cold-to-hot” converter

The preclinical evidence positions SSd as a multi-target network disruptor that simultaneously addresses the cellular, metabolic, and physical barriers defining “cold” HCC. Unlike single-mechanism agents that are often subverted by compensatory pathway activation, SSd’s coordinated modulation of PI3K/AKT/mTOR, STAT3, HIF-1α, and TGF-β signaling creates reinforcing effects: HIF-1α suppression reduces hypoxia and lactate accumulation, enhancing T-cell metabolic fitness; concurrent STAT3 inhibition reduces IDO1 expression, further relieving metabolic suppression; TGF-β attenuation reduces collagen deposition, improving T-cell physical access; and CXCL9/10 upregulation provides directional cues for recruitment. This network-level intervention aligns with the emerging recognition that durable immune remodeling in solid tumors requires simultaneous targeting of multiple suppressive nodes (120). These coordinated actions are summarized in **Figure 4**, which depicts the three principal immune-activating signaling axes of SSd.

**Figure 4:**
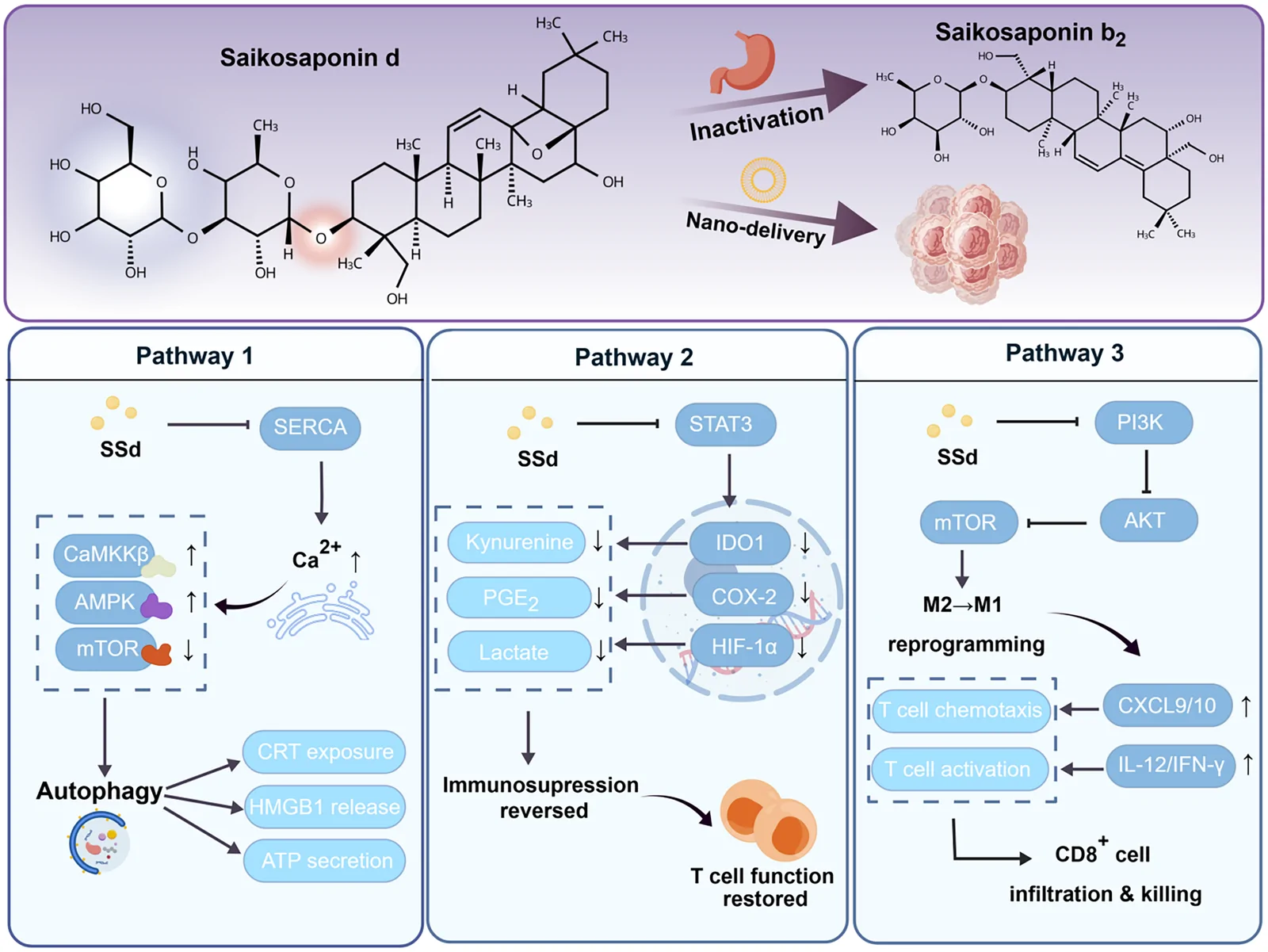
**(A)** Molecular structure of SSd and its metabolic degradation pathway in the digestive tract, which generates the inactive metabolite SSb_2_ and highlights the major obstacle to oral administration. Nano-delivery systems are proposed as a promising strategy to bypass this pharmacokinetic bottleneck. **(B)** Three proposed immune-activating signaling axes of SSd within the tumor microenvironment. First, SSd elevates cytosolic Ca²^+^ by inhibiting SERCA, which activates the CaMKKβ-AMPK-mTOR axis and induces autophagy-associated ICD. Second, SSd suppresses STAT3 signaling, leading to downregulation of IDO1, COX-2, and HIF-1α, which reduces immunosuppressive metabolites including kynurenine, PGE_2_, and lactate, thereby restoring T-cell function. Third, SSd inhibits the PI3K-AKT-mTOR pathway to repolarize macrophages from the M2 to the M1 phenotype, promoting the release of CXCL9/10 and IL-12/IFN-γ, which facilitates CD8^+^ T-cell infiltration and cytotoxicity. Pathway 1 and Pathway 2 are described in Section 5.3; Pathway 3 is detailed in Section 5.4.

The hepatic microenvironment, while presenting unique barriers, paradoxically enhances SSd’s therapeutic index. The liver’s continuous exposure to gut-derived antigens maintains resident immune populations in a partially activated state, potentially lowering the threshold for SSd-mediated immune conversion. Furthermore, the cirrhotic liver’s existing fibrotic infrastructure, while impeding T-cell infiltration (121), provides a targetable substrate for SSd’s anti-fibrotic effects (122). These liver-specific considerations suggest that SSd’s immunomodulatory efficacy may be amplified, rather than diminished, in the hepatic context compared to other solid malignancies.

### 5.6 Temporal dynamics: the four-stage process of immune conversion

Beyond the network-level and spatial considerations discussed above, a further layer of understanding emerges when these mechanisms are viewed through the lens of temporal progression. This perspective organizes them into a four-stage sequence, advancing from initial cellular stress signals to sustained immune remodeling, with each stage building upon its predecessor to form an interlocking cascade.

#### 5.6.1 Stage 1: initiation

We propose that, upon entering tumor cells, SSd engages SERCA and increases cytosolic calcium, activating autophagic flux via CaMKKβ/AMPK/mTOR signaling. This sequence has been observed in non-tumor and apoptosis-defective cells, but not in HCC. If operative in HCC, the resulting autophagic response could promote the core DAMP triad, CRT surface exposure, ATP secretion, and HMGB1 release, which together provide “find me” and “eat me” signals for subsequent immune activation (123). Simultaneously, SSd suppresses p-STAT3/HIF-1α signaling to downregulate COX-2 expression, attenuating the inherent immunosuppressive state of the TME. Through these coordinated effects, SSd could induce immunogenic stress signals within tumor cells while dismantling suppressive barriers at the microenvironmental level, establishing the preconditions for initiating the immune cascade. These predictions require experimental validation in orthotopic HCC models, with PK/PD endpoints as outlined in **Table 2**.

**Table 2:** Key validation gaps for clinical translation of SSd nanoformulations.

Phase	Functional node	Key validation gap	Proposed methodology
Stage I:Initiation	Intratumoral drug exposure	Define minimal immunostimulatory concentration in orthotopic HCC; validate Cmax/MIC >1 for nanoformulations.	LC-MS/MS quantification of SSd concentration in tumor homogenates; non-compartmental PK/PD modeling to establish Cmax/MIC ratio.
	Metabolic stability	Verify altered metabolite profile after encapsulation; compare metabolic fate of free vs. encapsulated SSd.	UPLC-Q-TOF-MS metabolomic profiling of plasma, liver, and tumor tissues; comparison of parent drug/metabolite ratios between free and encapsulated formulations.
Stage II: Amplification	ICD induction (CRT, ATP, HMGB1)	Perform gold-standard ICD triple assay; conduct prophylactic vaccination experiment.	Flow cytometry (CRT surface exposure); luminometric ATP assay; ELISA (HMGB1 release); tumor rechallenge in vaccinated mice to test immunologic memory.
	DC maturation	Validate tumor-infiltrating DC phenotype recovery in orthotopic HCC; assess cross-priming efficiency.	Multiplex immunofluorescence or flow cytometry (CD80/CD86/MHC-II/DC-LAMP on CD11c^+^ cells); ex vivo T-cell proliferation assay with CFSE labeling.
Stage III: Expansion	T-cell recruitment	Establish CXCL9 gradient in orthotopic HCC; track directional migration of CXCR3^+^ effector T cells.	Immunofluorescence or IHC for CD8/CXCR3/CXCL9 on serial tumor sections; laser capture microdissection followed by qPCR for CXCL9/10; flow cytometry for intratumoral CD8^+^CXCR3^+^ T cells.
	Cytotoxicity	Perform *in vitro* killing assay, *in vivo* tumor regression study, and dose-response evaluation in orthotopic HCC models; test synergy with immune checkpoint inhibitors.	Chromium-51 release assay or flow cytometry-based cytotoxicity assay; tumor growth curve and survival analysis in immunocompetent orthotopic HCC models; combination index (CI) calculation with anti-PD-1/PD-L1.
Stage IV: Consolidation	Macrophage polarization	Validate phenotype switch in Kupffer cells and infiltrating macrophages in orthotopic HCC; measure Arg-1 and iNOS activity.	Flow cytometry (CD206/CD86/iNOS/Arg-1 on F4/80^+^ cells); qPCR for M1 (iNOS, IL-12) and M2 (Arg-1, IL-10) markers; colorimetric Arg-1 activity assay; Griess assay for nitrite (iNOS activity).
	Metabolic normalization	Monitor dynamic lactate changes in orthotopic HCC; assess recovery of T-cell metabolic fitness.	Lactate quantification using enzymatic colorimetric assay on tumor interstitial fluid or homogenates; Seahorse XF analysis for T-cell OCR (oxidative phosphorylation) and ECAR (glycolysis) ex vivo.
	Physical barrier alleviation	Quantify collagen deposition in orthotopic HCC; evaluate matrix stiffness by atomic force microscopy.	Sirius red/fast green staining with digital image analysis; atomic force microscopy (AFM) or rheometry for tissue stiffness measurement; second harmonic generation microscopy for collagen fiber organization.
	Vascular normalization	Validate vascular structure and function in orthotopic HCC; assess T-cell transendothelial migration efficiency.	Immunofluorescence (CD31/α-SMA/pericyte coverage NG2 or PDGFR-β); lectin perfusion assay for vessel functionality; *in vitro* transwell TEM assay using HCC endothelial cells and primary CD8^+^ T cells.

#### 5.6.2 Stage 2: amplification

Once released, the DAMPs from Stage I would need to be captured by antigen-presenting cells to initiate the next wave of SSd-driven effects. Among these effects, the functional maturation of dendritic cells is a key predicted step. In human monocyte-derived DCs *in vitro*, SSd has been observed to upregulate co-stimulatory molecules and MHC-II, enhance IL-12 secretion, and increase the capacity to stimulate naïve CD8^+^ T-cell proliferation. If similar effects occur in HCC-infiltrating DCs, SSd-mediated inhibition of STAT3 phosphorylation could relieve maturation arrest and restore antigen processing and cross-presentation capacity. This would convert the initial DAMP signals from Stage I into a productive adaptive immune response.These predictions require experimental validation in orthotopic HCC models, with DC maturation and cross-priming endpoints as outlined in **Table 2**.

#### 5.6.3 Stage 3: expansion

We further propose that, once antigen-specific T-cell responses are initiated, SSd would promote Th1 polarization and IFN-γ production, which in turn induces CXCL9 and CXCL10 expression to recruit CXCR3^+^ effector T cells into the tumor parenchyma (124). Evidence for Th1 polarization derives from human mo-DC studies and from SSa in breast cancer models, but direct HCC data are lacking. Sustained PI3K/AKT/mTOR inhibition would be expected to reduce lactate accumulation and alleviates metabolic suppression of T-cell function (125, 126). Consistent with this, SSd has been shown to inhibit glycolysis and reduce lactate production in intrahepatic cholangiocarcinoma cells (127), suggesting that it may similarly attenuate lactate-driven T-cell suppression in HCC. SSd inhibits this pathway (128) and may thereby shift macrophages from an M2 to an M1 phenotype, mitigating myeloid-cell-associated immunosuppression.

A dose-dependent biphasic pattern is hypothesized but remains to be established. At lower concentrations, SSd promotes DC maturation and Th1 polarization without overtly dampening effector T-cell function. At higher concentrations, SSd inhibits T-cell activation through PKCθ, JNK, and NF-κB pathways (129). This non-monotonic response pattern suggests two distinct dose effects. Lower, sustained doses may prime antitumor immunity, while transient higher doses could promote immunogenic cell death without causing persistent T-cell suppression. The net outcome *in vivo* likely depends on the local concentration gradients achieved within the tumor microenvironment, a parameter that remains to be defined through systematic dose-escalation studies in orthotopic HCC models.These predictions require experimental validation in orthotopic HCC models, with T-cell recruitment, cytotoxicity, and dose-escalation endpoints as outlined in **Table 2**.

#### 5.6.4 Stage 4: consolidation

The final stage of this framework rests on the proposition that, once effector T cells are recruited to the tumor site, the durability of the antitumor response depends on whether these cells can maintain their functional state and form immunological memory. SSd’s inhibition of mTOR in hepatoma cells promotes autophagy and immunogenic cell death in HCC cells, yet T cell effector function and memory formation also require mTOR signaling. This apparent paradox might be resolved by spatial segregation: with tumor-targeted nanoformulations, SSd concentrations sufficient for mTOR inhibition are achieved predominantly within tumor cells, while T cells in the tumor microenvironment are exposed to lower, non-suppressive levels. This spatial uncoupling allows the beneficial effects of mTOR inhibition in tumor cells to be separated from its potential deleterious effects on T cell immunity. Sustained M1-like macrophage polarization would further support a pro-inflammatory milieu that maintains effector T cell function, establishing conditions for durable immune surveillance. These predictions require experimental validation in orthotopic HCC models, with macrophage polarization, metabolic normalization, and stromal/vascular remodeling endpoints as outlined in **Table 2**.

Collectively, these four stages are interlinked and sequentially amplified, constituting a hypothetical framework for SSd-mediated cold-to-hot immune conversion in HCC (**Figure 5**).

**Figure 5:**
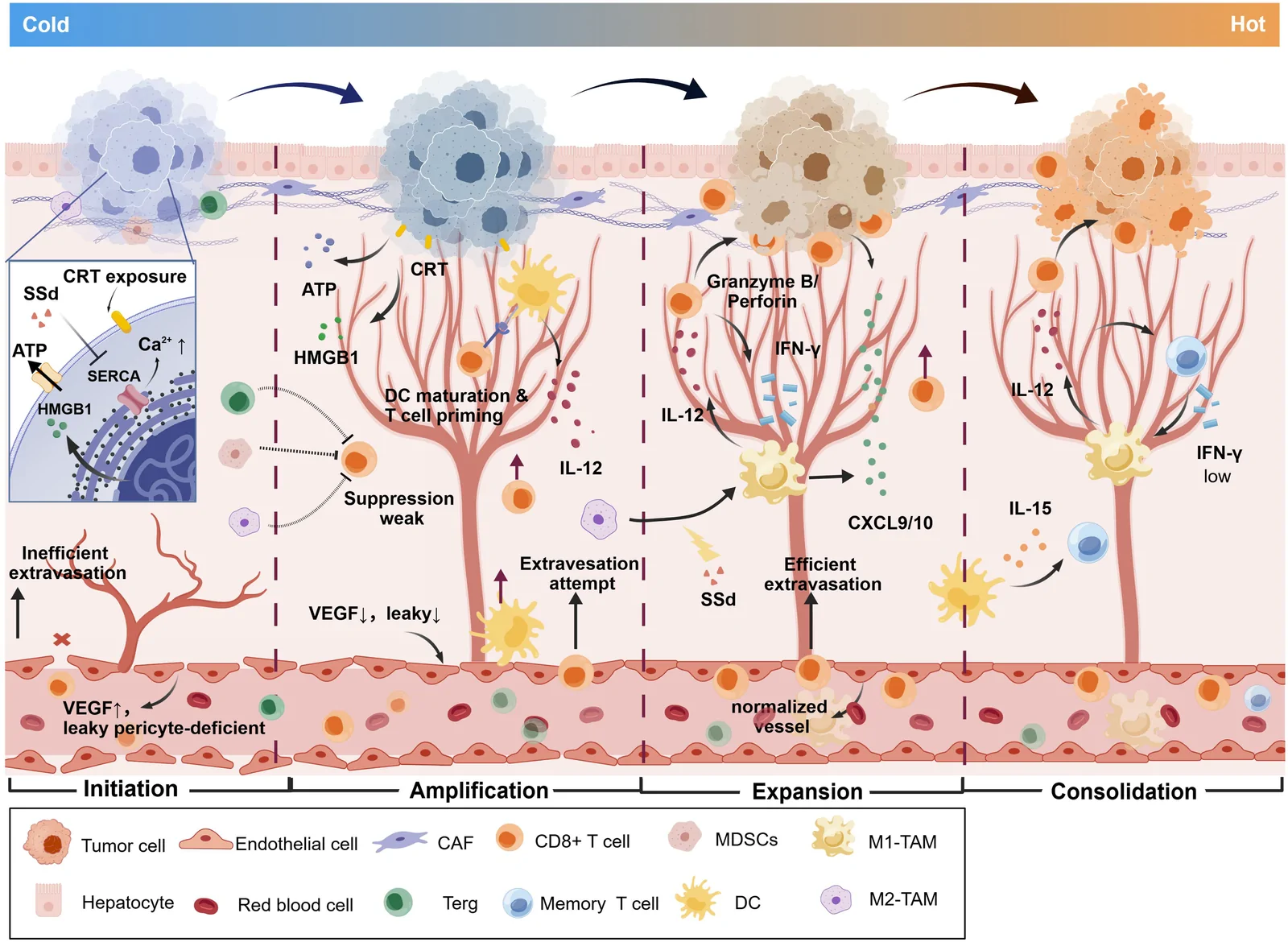
Four-stage temporal model of SSd-mediated cold-to-hot immune conversion in hepatocellular carcinoma.

### 5.7 Comparative positioning

The four-stage framework outlined above describes how SSd orchestrates immune conversion in theory. A comparison with existing therapeutic modalities further clarifies what distinguishes this framework from other approaches. Converting “cold” tumors to “hot” tumors requires more than isolated immune activation—it demands sustained engagement, physical access, and metabolic fitness Existing therapeutic modalities each excel at certain elements but fall short of full integration. TLR agonists such as imiquimod trigger rapid innate immune responses, yet these typically subside within days (130). Certain chemotherapeutic agents can induce immunogenic cell death, but their therapeutic window is narrow, and without appropriate immune co-stimulation, there is a risk of tolerogenic conversion (131). Immunomodulatory drugs (IMiDs) such as pomalidomide exert gradual modulation over weeks of continuous administration (132). Each of these classes achieves partial remodeling; none completes the full trajectory from initiation to sustained effector function.

SSd occupies a distinctive mechanistic niche. Its temporal architecture comprises four key processes: initiating autophagic cell death together with HIF-1α suppression, promoting dendritic cell maturation, establishing chemokine gradients, and consolidating immune memory. The biological relevance of these processes is supported by several lines of evidence. Autophagic and hypoxic responses have been linked to immune outcomes in various contexts (133, 134). DC maturation programs are regulated by pathways such as JAK2-STAT3, while chemokine gradients are central to immune cell trafficking. TGF-β-driven immune exclusion, moreover, is a well-established barrier to effective therapy (135). On the spatial side, SSd is hypothesized to dismantle physical barriers through TGF-β suppression and collagen reduction, and to normalize aberrant vasculature via HIF-1α inhibition. Although direct evidence for these effects in HCC is currently lacking, analogous effects have been observed with other agents in melanoma (136) and HCC (137), supporting the plausibility of such remodeling by SSd.

Beyond this spatiotemporal integration, SSd’s multi-target modulation of PI3K/AKT/mTOR, STAT3, HIF-1α, and TGF-β pathways creates network-level interactions that may limit compensatory escape observed with single-mechanism agents. Whereas other agents either trigger transient responses or modulate without ensuring immune access, SSd orchestrates comprehensive microenvironment remodeling. This mechanistic profile positions SSd not as a replacement for existing immunotherapies but as a priming agent, bridging immune ignition with sustained persistence while providing spatial infrastructure for checkpoint inhibitor efficacy. Its optimal role lies in microenvironment preparation: establishing chemokine gradients, opening physical corridors, and sustaining metabolic competence, so that subsequent immunotherapy encounters a receptive rather than a hostile terrain.

## 6 From mechanistic rationales to clinical hypotheses: SSd combination strategies

The multifaceted immunomodulatory properties of SSd suggest potential opportunities for combination with established cancer therapies. The following sections propose mechanistically plausible combination strategies informed by pathway complementarity and preclinical observations. None has been tested in HCC models, and thus these strategies remain hypothetical until validated.

### 6.1 Combination with chemotherapy: upgrading cytotoxicity to immunogenic cytotoxicity

#### 6.1.1 SSd + doxorubicin

In doxorubicin-resistant breast cancer models, SSd enhanced doxorubicin efficacy by inhibiting P-glycoprotein efflux and perturbing NQO1-mediated redox balance, increasing intracellular doxorubicin accumulation by 1.8-fold (138). The redox-targeting mechanism is plausibly conserved across cell types, but SSd+doxorubicin has not been tested in HCC. Given that doxorubicin remains a cornerstone of transarterial chemoembolization (TACE) in HCC, we propose evaluating this combination in HCC cell lines and, if positive, in the TACE setting with formal combination index calculations.

#### 6.1.2 SSd + platinum agents

Oxaliplatin induces immunogenic cell death in HCC cells and synergizes with anti-PD-1 therapy in syngeneic H22 models (139). SSd suppresses COX-2/PGE2 signaling and STAT3 phosphorylation in hepatoma cells, pathways implicated in chemoresistance and PD-L1 regulation. Whether SSd could further enhance oxaliplatin-induced ICD or mitigate resistance mechanisms such as chemotherapy-driven PD-L1 upregulation remains unexplored. We propose evaluating this combination in HCC cell lines with ICD triple assays (CRT/ATP/HMGB1) and, if positive, in orthotopic HCC models.

### 6.2 Combination with targeted agents: dual drive for vascular normalization and immune heating

#### 6.2.1 SSd + sorafenib

Sorafenib, a multikinase inhibitor targeting VEGFR and PDGFR, initially normalizes tumor vasculature but paradoxically increases intratumoral hypoxia over prolonged exposure, a well-documented resistance mechanism in HCC. Its immunomodulatory effects are modest, with limited objective response rates as a single agent. SSd suppresses HIF-1α in hepatoma cells, raising the hypothesis that it could counteract sorafenib-induced hypoxic rebound. Whether this combination improves T-cell infiltration or delays sorafenib resistance requires direct testing in HCC models.

#### 6.2.2 SSd + lenvatinib

Lenvatinib downregulates VEGFR signaling to promote macrophage-derived CXCL9 secretion and GZMK^+^ CD8^+^ T-cell infiltration in HCC. However, the recruited T cells may encounter a metabolically hostile and physically restrictive microenvironment that limits their effector function. SSd suppresses M2 macrophage polarization through p-STAT6 and PI3K/AKT/mTOR inhibition in murine pancreatic cancer models and promotes DC maturation in human mo-DCs *in vitro*. Whether these myeloid-reprogramming effects translate to enhanced lenvatinib efficacy in HCC remains untested. The mechanistic complementarity between lenvatinib-mediated recruitment and SSd-mediated functional empowerment is conceptually coherent, but direct experimental validation is required.

### 6.3 Combination with immune checkpoint inhibitors: priming the microenvironment

The most clinically relevant combination strategy involves co-administration of SSd with anti-PD-1/PD-L1 antibodies. The rationale for this approach is assembled from mechanisms observed in non-HCC or *in vitro* systems. SSd promotes DC maturation in human mo-DCs, deactivates TAMs in murine pancreatic cancer, and induces autophagic cell death associated with ICD. Collectively, these effects target the “cold” tumor phenotype that underlies primary resistance to ICIs. The kinetics of these effects suggest a theoretical priming-consolidation sequence in which SSd would first remodel the TME, followed by anti-PD-1/PD-L1 to sustain T-cell activity, though this temporal rationale has not been tested in HCC. **Table 3** summarizes the mechanistic rationales, evidence sources, and validation priorities for each combination discussed above.

**Table 3:** Proposed combination strategies: mechanistic rationales, evidence sources, and next steps.

Combination partner	Rationale	Evidence supporting the rationale	Supporting references	Validation status in HCC	Next step
Doxorubicin	SSd inhibits P-gp efflux and disrupts NQO1-mediated redox balance, increasing intracellular drug accumulation	Breast cancer models; pathways are conserved across cell types	(138)	Untested	Test SSd + doxorubicin in HCC cell lines; evaluate TACE compatibility
Oxaliplatin	SSd suppresses COX-2/PGE2 and STAT3, potentially mitigating PD-L1 upregulation	STAT3/COX-2 link in HCC; oxaliplatin induces ICD in HCC	(18, 139)	Untested	Determine whether SSd enhances oxaliplatin-induced ICD and reduces PD-L1
Sorafenib	SSd inhibits HIF-1α to counteract hypoxic rebound and preserve T-cell metabolic fitness	Sorafenib-induced hypoxia documented in HCC; SSd suppresses HIF-1α	(20)	Untested	Assess CD8^+^ T-cell infiltration and tumor hypoxia in SSd + sorafenib-treated HCC models
Lenvatinib	SSd suppresses M2 macrophage polarization, complementing lenvatinib-mediated T-cell recruitment	M2 suppression in pancreatic cancer; CXCL9 induction in HCC	(12, 137)	Untested	Quantify M1/M2 macrophage ratio in SSd + lenvatinib-treated orthotopic HCC
Anti-PD-1/PD-L1	SSd modulates TAM polarization, induces ICD, and promotes DC maturation to prime the TME	Each component mechanism shown separately in non-HCC or *in vitro* systems	(12, 75, 98)	Untested	Conduct sequential dosing experiment in immunocompetent HCC models (SSd first, then anti-PD-1)

## 7 Discussion and future perspectives

### 7.1 Mechanistic integration and biological significance

The preclinical evidence assembled in this review identifies a consistent pattern: SSd simultaneously engages multiple signaling nodes whose combined inhibition addresses distinct but interconnected barriers within the HCC microenvironment. Rather than acting through a single dominant pathway, this multi-node engagement creates a network-level effect that no single-target agent can reproduce. These activities converge on the relief of immune exclusion and restoration of T-cell effector function. This suggests that SSd acts at a systems level, not through a single linear pathway.

The significance of this systems-level mechanism is particularly evident when considering the hepatic microenvironment, where chronic inflammation and stromal rigidity normally limit immunotherapy responses. In this context, the same features that pose barriers to conventional agents become targetable substrates for SSd’s multi-node intervention.

Nevertheless, several caveats temper this optimistic framework. First, most of the immunomodulatory effects of SSd have been characterized in non-HCC models, primarily pancreatic, lung, and breast cancers, or *in vitro* systems that do not fully recapitulate the complex immunosuppressive milieu of HCC. Second, the extremely low oral bioavailability of SSd means that the concentrations required for immune effects are pharmacologically unattainable through oral administration. Third, whether the metabolites of SSd retain immunomodulatory activity remains unknown, introducing uncertainty about the true effector molecules *in vivo*.

### 7.2 Current limitations of the evidence base

#### 7.2.1 Lack of orthotopic HCC models

The most critical gap in the current literature is the absence of SSd efficacy data in orthotopic HCC models. Existing studies rely on subcutaneous xenografts or syngeneic non-hepatic tumors. Neither model recapitulates the liver’s unique immune architecture, including its fenestrated endothelium, constitutive lymphocyte surveillance, and chronic inflammatory context. In addition, the cirrhotic liver microenvironment introduces confounding DAMPs arising from persistent TLR4 activation, which may obscure tumor-specific immunogenic signals. Without orthotopic validation, claims of “cold-to-hot” conversion remain theoretical.

Future studies should therefore prioritize orthotopic HCC models, such as H22 or Hepa1–6 in immunocompetent C57BL/6 mice, to validate SSd-induced changes in T-cell infiltration, M1/M2 macrophage ratio, and tumor regression. These models better recapitulate the liver’s unique immune architecture and would provide the first direct evidence of SSd efficacy in an HCC-relevant immune microenvironment.

#### 7.2.2 Undefined pharmacokinetic-pharmacodynamic relationship

The theoretical framework presented herein rests on a critical assumption: that *in vitro* immune effects translate into *in vivo* tumor microenvironment remodeling. This assumption is challenged by the pharmacokinetic reality of SSd, as detailed in Section 3.4. Its negligible oral bioavailability, extensive hepatic first-pass metabolism, and rapid microbial biotransformation result in extremely low systemic exposure. Consequently, the intratumoral concentration threshold required for ICD induction, DC maturation, and macrophage reprogramming remains undefined. It is also unclear whether nanoparticle delivery can achieve this threshold without toxicity, whether encapsulation alters the metabolic fate of SSd, and what dosing schedule would best align with the temporal dynamics of immune conversion. Until these questions are resolved, the PK/PD basis of the proposed framework remains an assumption rather than an empirical foundation.

#### 7.2.3 Absence of formal synergy studies

No study has formally evaluated SSd in combination with HCC standard-of-care therapies using validated synergy models. The combination strategies in Section 6 are mechanistically plausible but lack experimental support. The entire combination section rests on untested assumptions about SSd’s interactions with chemotherapeutics, targeted agents, or immune checkpoint inhibitors. The potential for unexpected pharmacokinetic or pharmacodynamic interactions, whether synergistic, antagonistic, or neutral, remains entirely unexplored. Until such data are generated, these combination strategies remain mechanistic hypotheses awaiting validation and fall short of clinically actionable regimens.

### 7.3 Clinical translation challenges: the biphasic dose response

The biphasic behavior hypothesized in the temporal model carries direct translational implications: if low doses are immunostimulatory and higher doses are suppressive, then defining the therapeutic window becomes a prerequisite for clinical development. This requires systematic dose-escalation studies in orthotopic HCC models, with immune monitoring across multiple dose levels. The goal is to map the full dose-response curve for each immune endpoint, including DC maturation, T-cell infiltration, and macrophage polarization. Biomarker analysis would then be essential to establish the concentration range that maximizes immune activation while avoiding T-cell suppression. Serial measurements of serum CXCL9, intratumoral CD8^+^ T-cell density, and the M1/M2 macrophage ratio are particularly informative for this purpose. Until such data are generated, the biphasic response remains a mechanistic hypothesis rather than a dosing guide for clinical translation.

### 7.4 Future directions

#### 7.4.1 Patient-derived organoid models

HCC organoids that preserve patient-specific genetic alterations and incorporate immune components provide a platform for validating the “cold-to-hot” conversion mediated by SSd in a clinically relevant context. Such models can be used to explore several key questions, including whether SSd overcomes β-catenin-mediated immune exclusion, the impact of different viral etiologies (HBV, HCV, NASH) on SSd efficacy, and the identification of optimal combination partners in genotype-specific subgroups.

#### 7.4.2 Clinical translation pathway for SSd delivery systems

To bridge the pharmacokinetic chasm between preclinical efficacy and clinical feasibility, a tiered development strategy is proposed for SSd nanoformulations.

Phase I – Preclinical PK/PD validation. Hepatocyte-targeted liposomal SSd should be validated in orthotopic HCC models. Concurrent pharmacokinetic and pharmacodynamic modeling is required to establish the threshold intratumoral concentration needed for immune activation, defined as the ratio of maximum concentration to the minimum immunostimulatory concentration, or Cmax/MIC.

Phase II – Ex vivo efficacy comparison. Patient-derived HCC organoids should be employed to compare free SSd, nano-encapsulated SSd, and TACE-delivered SSd. The primary endpoint is the activation of the ‘cold-to-hot’ conversion signature, including DAMPs release, dendritic cell maturation, and CD8^+^ T cell cytotoxicity, as summarized in **Table 2**.

Phase III – Clinical trial design. A biomarker-driven basket trial combining SSd with anti-PD-1 antibodies is proposed for advanced HCC. Mandatory on-treatment biopsies must be collected for multiplex immunofluorescence to assess T cell infiltration and the M1/M2 macrophage ratio, and for liquid chromatography-tandem mass spectrometry to quantify intratumoral SSd concentration, thereby enabling direct PK/PD correlation in patients.

This stepwise framework aims to de-risk the clinical translation of SSd, transforming it from a hypothesis-generating agent into a clinically actionable immunomodulator.

## 8 Conclusion

This review positions SSd as a promising candidate for microenvironmental priming, with the potential to relieve key barriers that define immunologically “cold” HCC. Its multi-target profile addresses multiple nodes of the cancer-immunity cycle, including immunogenic cell death, dendritic cell maturation, T-cell recruitment, macrophage polarization, and metabolic and stromal remodeling.

Across the 29 primary studies on SSd, these mechanisms converge on core pathways that govern both HCC progression and immune regulation, laying a solid mechanistic foundation for the proposed “cold-to-hot” tumor immune conversion framework. To ensure full research transparency, we have systematically graded the quality of the included evidence and outlined a stepwise translational validation roadmap, while explicitly acknowledging that the majority of current findings are derived from preclinical experimental models. Translating these preclinical findings into clinical benefit will require overcoming several hurdles. The extremely low oral bioavailability of SSd, the absence of efficacy data in orthotopic HCC models, and the lack of a defined PK/PD relationship between *in vitro* immune effects and *in vivo* tumor remodeling are key challenges. Advanced delivery systems, particularly hepatocyte-targeted nanoparticles and transarterial formulations, offer a potential path to overcome bioavailability limitations. Similarly, although the mechanistic rationale for combining SSd with chemotherapeutics, targeted agents, and immune checkpoint inhibitors is coherent, formal synergy studies in HCC-relevant models are urgently needed.

The optimal role for SSd in HCC is likely not as a replacement for existing immunotherapies, but rather as a microenvironment-priming agent that prepares the tumor for checkpoint blockade. Realizing this potential will require interdisciplinary collaboration across medicinal chemistry, immuno-oncology, hepatology, and systems pharmacology. If forthcoming studies validate the proposed framework in orthotopic models and establish biomarker-guided patient selection strategies, SSd could expand the proportion of HCC patients who derive durable benefit from immunotherapy, addressing one of the most pressing unmet needs in liver cancer management.

## Glossary

AHR: Aryl hydrocarbon receptor
ADPKD: Autosomal dominant polycystic kidney disease
AMPK: AMP-activated protein kinase
ATP: Adenosine triphosphate
CAF: Cancer-associated fibroblast
Cmax: Maximum concentration
COX-2: Cyclooxygenase-2
CRT: Calreticulin
CTL: Cytotoxic T lymphocyte
CXCR3: C-X-C motif chemokine receptor 3
DAMPs: Damage-associated molecular patterns
DC: Dendritic cell
HCC: Hepatocellular carcinoma
HIF-1α: Hypoxia-inducible factor-1α
HMGB1: High-mobility group box 1
ICI: Immune checkpoint inhibitor
ICD: Immunogenic cell death
IDO1: Indoleamine 2,3-dioxygenase 1
IFN-γ: Interferon-gamma
IL-6/IL-10: Interleukin-6/Interleukin-10
iNOS: Inducible nitric oxide synthase
MDSC: Myeloid-derived suppressor cell
MHC: Major histocompatibility complex
MIC: Minimum immunostimulatory concentration
mTOR: Mammalian target of rapamycin
NF-κB: Nuclear factor kappa-light-chain-enhancer of activated B cells
NK: Natural killer
PDGFR: Platelet-derived growth factor receptor
PGE2: Prostaglandin E2
P-gp: P-glycoprotein
PI3K: Phosphoinositide 3-kinase
PK/PD: Pharmacokinetic/pharmacodynamic
PKB: Protein kinase B
PLGA: Poly(lactic-co-glycolic acid)
SERCA: Sarco/endoplasmic reticulum Ca2+-ATPase
SSd: Saikosaponin D
STAT3: Signal transducer and activator of transcription 3
TAM: Tumor-associated macrophage
TACE: Transarterial chemoembolization
TGF-β: Transforming growth factor-beta
Th1: T helper 1
TLR4: Toll-like receptor 4
TME: Tumor microenvironment
TNF-α: Tumor necrosis factor-alpha
Treg: Regulatory T cell
VEGF/VEGFR: Vascular endothelial growth factor/its receptor
