# Supplementary material for: From cold to hot: the immunomodulatory framework of Saikosaponin D in hepatocellular carcinoma therapy
Source: Front Immunol. 2026 Oct 1;17:1964907. doi: 10.3389/fimmu.2026.1964907 (PMC13670170; doi:10.3389/fimmu.2026.1964907)
Supplement: Supplementary file 1 [file Table1.docx]

**Supplementary Table S1:**

Complete search strategies used in each database (executed on 31 December 2025)

| **Database** | **Platform** | **Search strategy** | **Limits** |
| --- | --- | --- | --- |
| PubMed | NCBI | ("Saikosaponin D"[Title/Abstract] OR "SSd"[Title/Abstract]) AND (("hepatocellular carcinoma"[MeSH Terms] OR "HCC"[Title/Abstract] OR "liver cancer"[Title/Abstract] OR "liver neoplasm*"[Title/Abstract]) OR ("solid tumor*"[Title/Abstract] OR "solid tumour*"[Title/Abstract])) AND (immun*[Title/Abstract] OR "immune microenvironment"[Title/Abstract] OR immunomodulat*[Title/Abstract] OR "immunogenic cell death"[Title/Abstract] OR macrophage*[Title/Abstract] OR "T cell"[Title/Abstract] OR "dendritic cell"[Title/Abstract] OR "immune checkpoint"[Title/Abstract]) | English; peer‑reviewed |
| EMBASE | Elsevier | ('saikosaponin d'/exp OR 'ssd') AND (('liver cell carcinoma'/exp OR 'hepatocellular carcinoma'/exp OR 'hcc' OR 'liver cancer') OR ('solid tumor'/exp OR 'solid tumour')) AND (immun* OR 'immunomodulation'/exp OR 'immunogenic cell death'/exp OR macrophage* OR 't cell'/exp OR 'dendritic cell'/exp OR 'immune checkpoint'/exp) | English; peer‑reviewed |
| Web of Science Core Collection | Clarivate | TS=(("Saikosaponin D" OR "SSd")) AND TS=(("hepatocellular carcinoma" OR "HCC" OR "liver cancer" OR "liver neoplasm*") OR ("solid tumor*" OR "solid tumour*")) AND TS=((immun* OR "immune microenvironment" OR immunomodulat* OR "immunogenic cell death" OR macrophage* OR "T cell" OR "dendritic cell" OR "immune checkpoint")) | English; peer‑reviewed |
| Cochrane CENTRAL | Wiley | #1 MeSH descriptor: [Carcinoma, Hepatocellular] explode all trees  #2 (hepatocellular carcinoma OR HCC OR liver cancer):ti,ab,kw  #3 #1 OR #2  #4 (Saikosaponin D OR SSd):ti,ab,kw  #5 (immun* OR macrophage* OR T cell OR dendritic cell):ti,ab,kw  #6 #4 AND (#3) AND #5 | English; peer‑reviewed |
