# Supplementary material for: From cold to hot: the immunomodulatory framework of Saikosaponin D in hepatocellular carcinoma therapy
Source: Front Immunol. 2026 Oct 1;17:1964907. doi: 10.3389/fimmu.2026.1964907 (PMC13670170; doi:10.3389/fimmu.2026.1964907)
Supplement: Supplementary file 2 [file Table2.docx]

**Supplementary Table S2:**

Complete evidence matrix of the 29 primary SSd‑related studies included in this review.

| **Ref** | **Model system (cell line/animal/species)** | **SSd dose/concentration & route** | **Key immune‑related finding** | **Evidence grade** | **Key limitation** |
| --- | --- | --- | --- | --- | --- |
| (12) | Murine orthotopic pancreatic cancer model (PDAC); in vitro: Panc02 and H7 cells, BM-MNCs, RAW 264.7 macrophages | In vitro: IC₅₀ = 7.879 μM (Panc02) and 15.69 μM (H7) | Decreased M2 macrophage polarization; downregulated p‑STAT6 and PI3K/AKT/mTOR signaling; reactivated local immune response | I‑nonHCC | In vitro only; non‑immune endpoint |
| (13) | Human HCC cell lines Hep G2 (p53⁺/Fas⁺) and Hep 3B (p53⁻/Fas⁻) | In vitro: 1, 2.5, 5, 10 μM (24–48 h); IC₅₀ = 2.63 μM (Hep G2), 4.26 μM (Hep 3B) at 48 h | Induced G0/G1 arrest in Hep G2 via p53/p21/WAF1; induced apoptosis in both lines; upregulated Fas/APO-1, mFasL, sFasL and Bax (Hep G2); enhanced IκBα, decreased nuclear NF-κB and Bcl-XL; effects partially blocked by anti-Fas ZB4 | D-HCC | In vitro only; non-immune endpoint (apoptosis/cell cycle) |
| (14) | Human HCC cell lines (PLC, MHCC‑97H, SMMC‑7721, Huh7) | In vitro, 48h: IC₅₀ = 39.87–51.48 μmol·L⁻¹ | Induced autophagic cell death via negative regulation of mTORC pathway; upregulated LC3‑II and Beclin 1; downregulated p‑S6K1 | D‑HCC | In vitro only; no immune readout |
| (18) | Human HCC cell line SMMC‑7721 (hypoxia, combined with radiotherapy) | In vitro, hypoxic condition: 2.5, 5.0, 10.0, 15.0 μg/mL | Enhanced radiosensitivity under hypoxic conditions via HIF‑1α inhibition | D‑HCC | Radiosensitization; non‑immune endpoint; in vitro only |
| (19) | Human HCC cell lines (SMMC‑7721, HepG2) | In vitro, 24-72h: 2.5, 5.0, 10.0, 15.0 μg/mL | Suppressed COX‑2 via p‑STAT3/C/EBPβ pathway; inhibited cell proliferation in a dose‑dependent manner | D‑HCC | In vitro; immune cells not examined |
| (20) | Human HCC cell line SMMC‑7721 (normoxia and hypoxia, combined with 2 Gy radiotherapy) | In vitro: 1, 3 μg/mL (2 h prior to irradiation) | Enhanced radiosensitivity; induced G0/G1 arrest and decreased S‑phase population; reduced G2/M‑phase population under hypoxia; upregulated p53 and BAX; downregulated Bcl‑2 | D‑HCC | Radiosensitization; non‑immune endpoint; in vitro only |
| (22) | Human L-O2 hepatocytes; male ICR mice | In vitro: 0.8, 1, 2 μM (24 h); In vivo: 300 mg/kg, oral gavage, once daily for 1 week | Hepatotoxicity: Fas-mediated extrinsic apoptosis via caspase-8 cleavage and Bid activation, leading to cytochrome c release and caspase-3 activation; in vivo liver/body weight ratio increased, massive hepatocyte apoptosis, Bcl-2 downregulated and Bax upregulated | D-HCC | Non-HCC; normal hepatocytes and mice, not HCC models |
| (23) | Sheep red blood cells (SRBC) | In vitro: 1.0–5.0 μg/mL | Hemolysis: dose-dependent lysis of erythrocytes; hemolytic activity correlated with adsorbability on red blood cell membranes | I-IV | Non-tumor; in vitro only |
| (24) | Cardiomyocytes and hepatocytes | In vitro: equivalent concentrations of SSa and SSd | Cardiotoxicity and hepatotoxicity: SSa and SSd show higher affinity for SERCA and stronger toxic responses on cardiomyocytes and hepatocytes than other saikosaponins | I-IV | Non-tumor; in vitro only |
| (25) | Caco‑2 cell monolayers (in vitro) and rat single‑pass intestinal perfusion model (in vivo) | 0.5–5 μmol/L (concentration‑dependent transport, 0–180 min) | Intestinal absorption mechanism: low‑permeability compound; P‑gp and paracellular pathway involved; ileum as main absorption site | I‑IV | Non‑immune; non‑tumor; pharmacokinetic study only |
| (26) | SD rats | In vivo: 5 mg/kg (i.v.) or 50, 100, 200 mg/kg (p.o.) | Oral bioavailability ~0.04%; i.v. t₁/₂ = 2.29 h with hemolysis; dose‑dependent systemic exposure | I‑IV | SSa (epimer), not SSd; non‑immune; non‑tumor; pharmacokinetic study only |
| (50) | Human ADPKD cell line OX161; normal renal epithelial UCL93; MDCK cysts (3D collagen) | In vitro: 5.0 μM (4–24 h); 2.5–7.5 μM (7 days, cyst formation) | Inhibited proliferation (dose‑dependent cyst suppression); induced G0/G1 arrest (↑P27); as SERCA inhibitor, elevated cytosolic Ca²⁺, activated CaMKKβ‑AMPK, inhibited mTOR, induced autophagy (↑GFP‑LC3 puncta, ↑LC3B‑II, ↓p62); effects blocked by 3‑MA, Compound C, STO‑609, BAPTA/AM | I‑IV | Non‑tumor, non‑HCC (ADPKD); weakest extrapolation |
| (61) | Rat hepatic stellate cell line HSC‑T6 | In vitro: 0.01–5 μmol/L (24 h pretreatment + 4 h H₂O₂) | Suppressed oxidative stress‑induced HSC proliferation and activation via ERβ; reduced TGF‑β1, Hyp, COL1, TIMP‑1; increased MMP‑1; downregulated ROS/MAPK | D‑HCC | Anti‑fibrotic; not directly immune endpoint |
| (69) | Human HCC cell lines SMMC‑7721 and MHCC97L (combined with radiotherapy) | In vitro: 3 μg/mL (2 h prior to irradiation) | Promoted autophagy via inhibiting mTOR phosphorylation; increased radiation‑induced apoptosis; upregulated LC3‑II and Beclin‑1; decreased p62; enhanced cleaved caspase‑3 and PARP; effects reversed by CQ, MHY1485, siRNA‑P53 or Compound C | D‑HCC | In vitro; no orthotopic/immune data |
| (75) | Human monocyte‑derived DCs (mo‑DCs) from condyloma patients | In vitro: 5, 10, 20 μM (5 days for differentiation, 48 h for maturation) | Inhibited monocyte‑to‑DC differentiation (downregulated CD1a/CD80/CD86, upregulated CD14); enhanced endocytic activity (CD32/MR, FITC‑dextran uptake); promoted DC maturation (upregulated CD40/CD83/CD80/CD86); enhanced IL‑12 secretion and CD4⁺ T‑cell proliferation | I‑IV | Non‑HCC (human DCs); in vitro; samples from condyloma patients |
| (86) | SD rat breast cancer model (DMBA‑induced) | In vivo: 35 mg/kg (i.g., once daily for 56 days) | Inhibited tumor growth (IR 62.84%); increased intratumoral CD8⁺ and CD4⁺ T‑cell infiltration; shifted Th1/Th2 balance toward Th1 (↑IFN‑γ, ↑IL‑12; ↓IL‑4, ↓IL‑10); activated IL‑12/STAT4 pathway | I‑nonHCC | SSa (epimer), not SSd; non‑HCC (breast cancer) |
| (87) | Mouse liver fibrosis model (CCl₄‑induced, WT, αERKO, βERKO mice); HSCs‑LX2 cells | In vivo: 2 mg/kg (s.c., twice weekly for 6 wk); in vitro: 5 μM (HSCs‑LX2, 24 h) | Alleviated CCl₄‑induced liver fibrosis via ERβ pathway; reduced α‑SMA, TGF‑β1, TIMP‑1, MMP‑2, Vimentin; increased E‑cadherin; decreased ROS and NLRP3 inflammasome proteins (NLRP3, pro‑IL‑1β, IL‑1β, IL‑18) | I‑nonHCC | Anti‑fibrotic; not directly immune endpoint |
| (88) | Rat liver fibrosis model (CCl₄ + alcohol + high‑fat/low‑protein diet, SD rats) | In vivo: 1.0, 1.5, 2.0 mg/kg (i.p., once daily for 6 wk) | Attenuated CCl₄‑induced hepatic fibrosis; reduced serum ALT, TG, HA, LN; decreased hepatic TNF‑α, IL‑6, HYP; downregulated NF‑κBp65; upregulated I‑κBα | I‑nonHCC | Anti‑fibrotic; non‑immune endpoint |
| (98) | Multiple cell lines (HeLa, MCF‑7, H1299, HepG2, MEFs) | In vitro: 10 μM (autophagy induction); LC₅₀ = 4.79–25.1 μM across cell lines | Novel SERCA inhibitor; increased cytosolic Ca²⁺ via SERCA inhibition; activated CaMKKβ‑AMPK‑mTOR pathway; induced autophagic cell death in apoptosis‑defective cells | I‑IV | Non‑HCC; mechanistic extrapolation; includes non‑tumor cells (MEFs, HeLa) |
| (99) | Human HCC cell lines SMMC‑7721 and MHCC97L (combined with 2 Gy radiotherapy) | In vitro: 3 μg/mL (2 h prior to irradiation) | Enhanced radiosensitivity by inducing autophagy formation; increased radiation‑induced autophagosomes and LC3‑II levels; effects reversed by chloroquine or MHY1485 | D‑HCC | In vitro; no orthotopic/immune data |
| (105) | Female C57BL/6N mice (6–8 wk), HT model induced by pig thyroglobulin + adjuvant | In vivo: 5 mg/kg (i.p., every other day for 6 wk) | Attenuated thyroid lymphocytic infiltration (P=0.044); reduced serum TPOAb (P<0.001); shifted M1/M2 balance toward M2 (P=0.003); inhibited Th1 (IFN‑γ) and Th17 (IL‑17) cytokines | I‑nonHCC | Non‑HCC (autoimmune thyroiditis model); not tumor microenvironment |
| (110) | Mouse liver fibrosis model (CCl₄‑induced, C57BL/6J); LX‑2 cells; primary mouse HSCs | In vivo: 2.0 mg/kg (i.p., daily for 6 wk); in vitro: 5 μM (LX‑2, 24 h with TGF‑β) | Alleviated CCl₄‑induced liver fibrosis (↓collagen, Col1α1, α‑SMA); inhibited NLRP3 inflammasome activation (↓NLRP3, IL‑18, IL‑1β); restored TGF‑β‑induced ERβ downregulation; effects partially blocked by THC (ERβ antagonist) | D‑HCC | Anti‑fibrotic; not directly immune endpoint |
| (111) | Rat hepatic stellate cell line HSC‑T6 | In vitro: 1 μM (ED₅₀ for apoptosis/migration assays; 0–3 μM for dose‑response, up to 72 h) | Inhibited HSC‑T6 proliferation and migration in a time‑ and dose‑dependent manner; induced apoptosis; decreased ERK1/2, PDGFR1, TGF‑β1R, α‑SMA, TGF‑β1, CTGF; decreased p‑p38 and p‑ERK1/2; blocked PDGF‑BB‑ and TGF‑β1‑induced proliferation and migration | D‑HCC | SSa+SSd comparison; non‑immune; anti‑fibrotic |
| (112) | Human NSCLC cell lines A549 and H1299 | In vitro: IC₅₀ = 3.57 µM (A549), 8.46 µM (H1299) | Dose‑dependently inhibited proliferation; induced G0/G1 arrest and apoptosis; downregulated p‑STAT3 and p‑ERK; upregulated cleaved caspase‑3 | I‑nonHCC | Non‑HCC (lung cancer); in vitro only |
| (113) | Human TNBC cell line MDA‑MB‑231 | In vitro: IC₅₀ = 7.293 µM (24 h); 2, 4, 6 µM for functional assays (24 h) | Decreased cell viability; inhibited colony formation and migration; induced morphological changes; modulated apoptotic (↑Bax, Bid, Caspase9/3; ↓Bcl2) and necroptotic (↑RIPK1/3, MLKL) markers; dose‑dependently inhibited pSTAT3 and c‑Myc expression | I‑nonHCC | Non‑HCC (breast cancer); includes in silico analysis; in vitro only |
| (121) | SD rat liver fibrosis model (DMN‑induced); CRL‑1548 rat hepatoma cells | In vivo: 1.8 mg/kg (i.p., daily for 4 wk); in vitro: 2 μg/mL (48 h pretreatment) | Reduced collagen I deposition; decreased serum ALT; decreased hepatic TGF‑β1; alleviated CCl₄‑induced hepatocyte oxidative injury (↓LDH release, ↓MDA production) | D‑HCC | Anti‑fibrotic; not directly immune endpoint |
| (127) | Human iCCA cell lines RBE and HuCCT1 | In vitro: 2.5, 5.0 μM (24–96 h, combined with gemcitabine) | Reversed NE/E‑induced gemcitabine resistance via downregulating ADRB2; inhibited drug efflux (MDR1, ABCG2) and glycolysis (HK2, GLUT1); reduced glucose consumption and lactate production | I‑nonHCC | Cholangiocarcinoma ≠ HCC; resistance mechanism, not immune |
| (128) | Human ESCC cell lines Eca‑109 and Te‑10 | In vitro: 0–15 μM (24 h); IC₅₀ = 11.53 μM (Eca‑109), 9.52 μM (Te‑10) | Network pharmacology predicted 31 intersecting targets, 5 core targets (EGFR, STAT3, SRC, ESR1, CASP3), 94 pathways (mainly PI3K‑AKT); molecular docking confirmed binding to 5 core targets (EGFR: −8.7 kcal/mol); inhibited proliferation, migration, invasion; promoted apoptosis; concentration‑dependently inhibited PI3K and p‑AKT expression | I‑nonHCC | Non‑HCC (esophageal cancer); includes in silico analysis (network pharmacology + molecular docking); in vitro only; no in vivo validation |
| (129) | Primary mouse T cells (lymph node, >97% purity) | In vitro: 5, 10, 20 μM (non‑cytotoxic; 20–48 h for surface markers; 40 h for IL‑2) | Dose‑dependently suppressed CD69 (early) and CD71 (late) expression in Con A‑ or PMA‑stimulated T cells; inhibited PMA‑induced PKCθ translocation; inhibited IκBα and JNK phosphorylation (not ERK); inhibited PMA/ionomycin‑stimulated IL‑2 production | I‑IV | Sole source of “inhibitory” biphasic dose data; in vitro only; primary mouse T cells, not human |
